# Open-label placebos for menopausal hot flushes: a randomized controlled trial

**DOI:** 10.1038/s41598-020-77255-z

**Published:** 2020-11-18

**Authors:** Yiqi Pan, Ramona Meister, Bernd Löwe, Ted J. Kaptchuk, Kai J. Buhling, Yvonne Nestoriuc

**Affiliations:** 1 Department of Clinical Psychology, Helmut-Schmidt-University/University of the Federal Armed Forces, Hamburg, Germany; 2 Department of Psychosomatic Medicine and Psychotherapy, University Medical Centre Hamburg-Eppendorf, Hamburg, Germany; 3 Department of Medical Psychology, University Medical Centre Hamburg-Eppendorf, Hamburg, Germany; 4 Program in Placebo Studies and the Therapeutic Encounter (PiPS), Beth Israel Deaconess Medical Center, Harvard Medical School, Boston, USA; 5 Department of Gynecological Endocrinology, Clinic of Gynecology, University Medical Centre Hamburg-Eppendorf, Hamburg, Germany

**Keywords:** Medical research, Outcomes research

## Abstract

This study investigated the efficacy of an open-label placebo (OLP) treatment for menopausal hot flushes. Women with at least five moderate or severe hot flushes per day were allocated to receive four weeks of OLP for twice a day or no-treatment. Intention-to-treat analyses included n = 100 women. In comparison to no-treatment, OLP reduced the log-transformed hot flush composite score (frequency × intensity) (mean difference in change: − 0.32, 95% CI [− 0.43; − 0.21], *p* < 0.001, Cohen’s* d* = 0.86), hot flush frequency (− 1.12 [− 1.81; − 0.43], *p* = 0.02, Cohen’s* d* = 0.51), and improved overall menopause-related quality of life (− 2.53 [− 4.17; − 0.89], *p* = 0.02, Cohen’s* d* = 0.49). Twelve (24%) (vs. three [6%]) patients had 50% lesser hot flushes. Problem rating of hot flushes and subdomains of quality of life did not improve. After four weeks, the OLP group was further divided via randomization to continue or discontinue the treatment. Benefits were maintained at week 8 (log-transformed score: − 0.04 [− 0.06; 0.14], *p* = 0.45). There was no difference between taking placebos for 8 or 4 weeks (log-transformed score: 0.04 [− 0.17; 0.25], *p* = 0.73). Results indicate that open-label placebos may be an effective, safe alternative for menopausal hot flushes.

## Introduction

Placebos are commonly used in clinical practice deceptively^1,2^. In a systematic review, Fässler and colleagues reported that 17–80% of clinicians administer placebos in such an ethically problematic manner^1^.

A growing body of studies demonstrated that placebos without deception can have beneficial effects. Prescribing placebos in an honest manner would eliminate the ethically problematic use of deceptive placebos or active medications that have no efficacy for the treated condition (“impure placebos”) in the clinical setting^2^. The first randomized controlled trial (RCT) on open-label placebos (OLP) was conducted in 2010 among patients with irritable bowel syndrome^3^, followed by further RCTs on chronic back pain^4,5^, depression^6^, allergic rhitinis^7^, and cancer-related fatigue^8^. A meta-analysis from 2017 found an overall positive effect of 0.9, indicating a large effect of OLP compared to no-treatment^9^.

Currently, these studies do not allow definite clinical conclusions because of their small sample size. Nonetheless, the end goal of OLP research is to improve patient care, especially for patients whose treatment options are limited, and when OLP has the potential to fill in the gaps in care.

One such patient group is women suffering from hot flushes. Hot flushes are the most commonly reported symptoms in menopause, occurring in up to 80% of all women and affecting quality of life to various degrees^10,11^. Women who experience hot flushes at night (also called night sweats) additionally suffer from lack of sleep, which in turn negatively impacts their functioning during the day. For decades, hot flushes have been treated successfully with hormone substitute therapy^12,13^. However, the Women’s Health Initiative (WHI) trial from the year 2002, investigating over 16,000 women taking hormone therapy, documented increased risks of adverse events, including breast cancer and stroke^14^. Since then, many clinicians and patients are seeking for non-hormonal treatment options. Although almost two decades have passed, the topicality of the WHI study remains as the association between breast cancer risk and hormone therapy has been recently validated in a meta-analysis using epidemiological data^15^.

To treat hot flushes, patients may choose from a large variety of complementary and alternative medicines, e.g., black cohosh, phytoestrogens, wild yam, Chinese herbal medicine, etc. Herbal-based remedies were researched extensively with meta-analyses or systematic reviews demonstrating mixed or no effects over placebo^16–18^. Likewise, mind–body interventions, including yoga, acupuncture, paced breathing, and relaxation appear ineffective in reducing hot flushes^13,19^. Selective serotonin reuptake inhibitors (SSRIs) and serotonin-norepinephrine update inhibitors (SNRIs), gabapentin, and clonidine are effective yet may cause side effects or withdrawal symptoms^20^. Given their safety profile, the benefits of these medications remain debatable^21,22^. Early studies have shown that cognitive-behavioural therapy and hypnosis may help^23,24^. However, the body of evidence is small.

Importantly, placebo effects in double-blind clinical trials are substantial. The mixed effectiveness of multiple remedies and large placebo effects across studies indicate that non-specific effects and related mechanisms such as expectations may play an important role^25^. In placebo arms, hot flushes were reduced by an average of 58% in hormone therapy trials^26^, and by 20–50% in non-hormonal trials^20^. Using pooled data from two studies, Freeman found that 33% of the women report half the hot flushes they had at baseline after taking eight weeks of placebos, indicating clinically meaningful improvements^25^. The magnitude of the placebo response may thus explain the inefficacy of some active treatments^27^. However, placebo effects in RCTs are confounded by natural history and statistical artefacts like regression to the mean since untreated groups are rarely included^28^. A no-treatment control can be important to separate placebo effects from these other confounders.

In this study, we investigate the efficacy of placebos without deception for menopausal hot flushes by comparing the OLP group with a no-treatment group. To further our understanding of the duration of OLP effects and to explore potential exposure–response relationships, we compare 4 and 8 weeks of placebo intake. Since its underlying mechanisms is unclear, we will additionally examine the link between expectations, hope, and optimism and OLP response. Whereas expectations are one of the most robust predictors of placebo response in laboratory studies^29^, optimism, and hope were additionally proposed as pertinent factors to OLP^30^, thus warranting scrutiny.

## Results

### Participant flow

**Figure 1:**
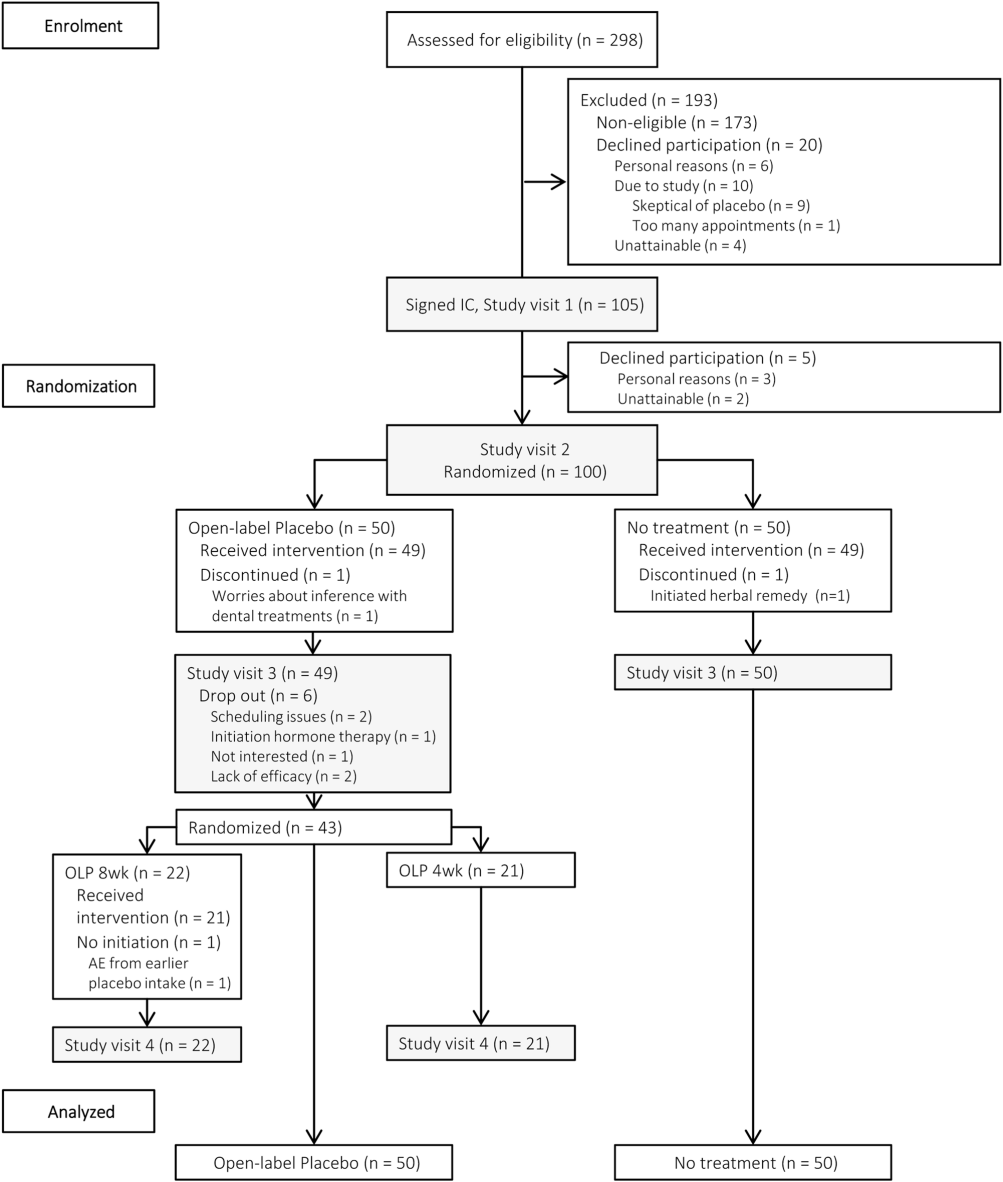
Flow of participants. *IC *informed consent, *OLP *open-label placebo, *wk *week, *AE *adverse event.

We randomized and analysed n = 100 participants (Fig. 1). One group knowingly received 4 weeks of placebos (OLP), whereas the other group received no treatment. Two patients (one in each group) dropped out (2%) during the treatment phase. After 4 weeks of treatment, six patients in the OLP group refused to participate in the follow-up phase, two of which did so due to unrelieved symptoms. One patient rejected further placebo intake yet completed diary and questionnaires at study visit 4. As a result, n = 22 and n = 21 patients were randomized to the OLP 8 week and OLP 4 week group.

### Recruitment

Participants were recruited in and around Hamburg, Germany, from the general population. We recruited from October 2018 to December 2019 and followed up from November 2018 to March 2020. Women were informed via leaflets at their practitioner (gynaecologists, general practitioners, practitioners of alternative medicine), pharmacies, stores, cultural institutions, and gyms. We also used online and newspaper advertising, and the internal newsletter of the University Medical Centre (UMC) Hamburg. This study was advertised as a “novel holistic treatment” (short leaflet) or as “open-label placebo treatment for hot flushes” (detailed leaflet, study website). Women contacted the study team via phone or email. The screening interview was standardized and included a stringent decision tree based on the eligibility criteria. Study assistants conducted it over the phone. If eligible, a first study visit was scheduled. All patients were explicitly informed about the study’s objective of testing honestly prescribed placebos with informed consent.

### Baseline characteristics

Patient characteristics are shown in Table 1. Most patients (76%) were post-menopausal, in a partnership (52%), and employed (75%). Time since onset of hot flushes varied between 30 days and 26 years (*M* = 5.2 years, *SD* = 5.4). Six patients had a history of breast cancer, of which four completed or discontinued endocrine therapy for at least one year. The majority had used some kind of hot flush treatment before (75%). No woman utilized body-mind interventions (e.g., yoga, meditation, hypnosis) for hot flushes. The number of daily hot flushes varied between 3.1 and 24.4. Baseline characteristics did not differ between the groups.

**Table 1:** Patient characteristics.

	OLP (n = 50)	No-treatment (n = 50)
Age (M, range)	54.2 (44–76)	54.9 (46–71)
**Marital status (N)**		
In a partnership	27	25
Single	17	15
Divorced	6	7
Widowed	0	3
**Highest education (N)**		
Middle school	22	17
High school	15	15
University	13	18
**Employment status (N)**		
Employed	35	40
Unemployed/home maker	8	6
Retired	7	4
**Menopausal status (N)**^a^		
Transition period (interval of amenorrhea ≥ 60 days)	14	10
Early post menopause (2–6 years since FMP)	33	35
Late post menopause (remaining life span)	3	5
**Years since onset of hot flush (N)**		
< 1 year	7	7
1–2 years	6	8
2–4 years	15	17
4–6 years	6	7
> 6 years	16	11
**Gynaecological history (N)**		
History of breast cancer	1	5
Hysterectomy	7	8
**Numbers of previous hot flush treatment (N)**		
None	16	9
1–3	30	36
4 or more	4	5
**Class of previous hot flush treatment**^b^** (N)**		
Black cohosh	18	20
Hormone therapy	5	10
Homeopathic remedy	8	7
Sage	5	8
Siberian rhubarb roots	4	3
Other^c^	13	21
**Body Mass Index, categorized (N)**^d^		
Underweight	4	1
Normal weight	19	29
Pre-obesity	14	15
Obese	13	5
**Smoking status**		
Never	23	18
Current	11	14
Former smoker	16	18
**Physical activity**		
Never	4	4
1–3 days per month	12	7
1–2 days per week	24	23
3 + days per week	10	16
**Number of hot flushes per day**^e^		
3–5	11	10
6–7	10	7
8–10	17	17
11+	12	16

*OLP *open-label placebo, *FMP *final menstrual period.

^a^Classified after the Stages of Reproductive Aging Workshop (STRAW) criteria.

^b^Numbers do not sum to 100 since multiple entries were possible.

^c^Other treatments include bioidentical hormones, chaste berry, citric acid, dandelion, devil’s claw, evening primrose, hemp seeds, Iceland moos, lady’s mantle, linseeds, pollen extract, pomegranate, red clover, soy, St. John’s wort, and wild yam.

^d^Classified after the World Health Organization.

^e^Based on the diary. Eligibility criteria required patients to have at least five hot flushes a day yet mean hot flushes during the baseline week might have differed from reports at the screening.

### Missing values

Single missing values in questionnaires and the diary were indicated by 25 and 18 patients, respectively. Missing values (per patient) ranged from 0.4 to 1.6% in questionnaires and from 0.7 to 5% in the diary. Except for one person who overlooked one page (6.7% missing values; not outcome-related), missing items were scattered across the questionnaires. At least 80% of the items were present for all scales. Missing data due to dropouts were very low (n = 1; 1%).

### Efficacy analyses

**Figure 2:**
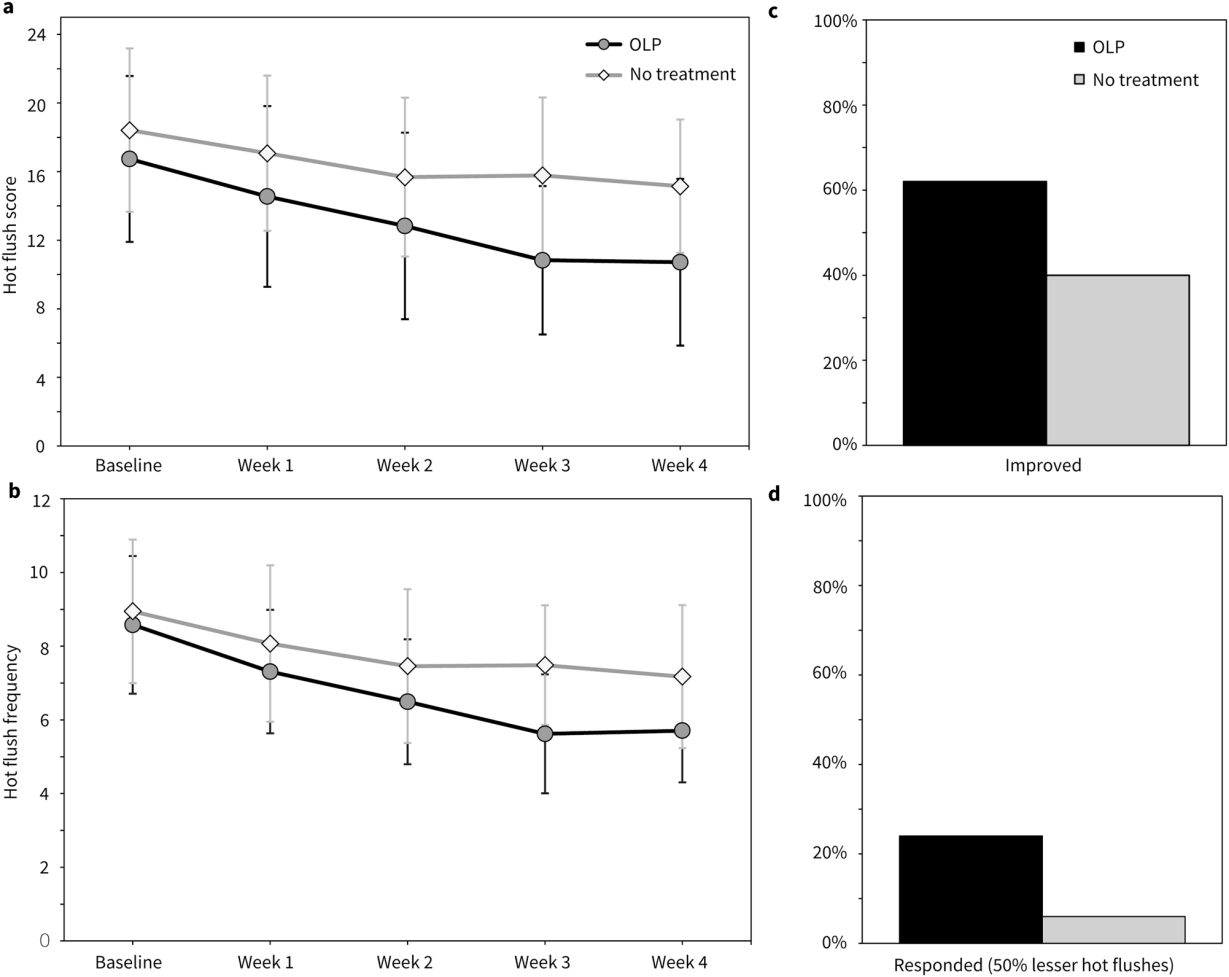
Changes in hot flushes and overall improvement (observed values). Changes in (**a**) hot flush score, and (**b**) hot flush frequency over time. (**c**) Proportion of patients who indicated improvement, and (**d**) proportion of patients with clinically meaningful improvements. Error bars indicate standard deviations. *OLP *Open-label placebo.

The OLP group reported a hot flush score of *M* = 16.74 (*SD* = 9.68) at baseline, and of *M* = 10.72 (*SD* = 9.73) at week 4. The no-treatment group reported a score of *M* = 18.41 (*SD* = 9.53) at baseline, and of *M* = 15.15 (*SD* = 7.78) at week 4 (Fig. 2). In comparison to no-treatment, OLP reduced the log-transformed hot flush score and hot flush frequency with effect sizes of Cohen’s *d* = 0.86 and *d* = 0.51, respectively, indicating large and moderate effects (Table 2). Back-transformed estimates of the hot flush score reduced by 6.65 points (43%) (vs. 2.99 points [19%]) in the OLP group. The onset of action appeared to be rapid, given that 40% was reduced after three weeks of intake. By week 4, estimates indicated that the OLP group had 2.90 lesser hot flushes (33%) (vs. 1.77 [20%]). Also, 31 (vs. 20) women reported to have overall improved (*χ*^2^ (1, *N* = 50) = 4.82, *p* = 0.03, Cramer’s *V* = 0.22). Twelve women (vs. 3) obtained a clinically relevant reduction by 50% in hot flush frequency (Fisher’s Exact Test: *p* = 0.02, Cramer’s *V* = 0.25) (Fig. 2). One case in the OLP group was identified as a multivariate outlier as she had a baseline score and frequency of 4.1 and 4.0 SD above the sample mean. After deleting this case, the results were identical to ITT. However, the effects were marginally larger (log-transformed hot flush score: − 0.33, 95% CI [− 0.45; − 0.21], *p* < 0.001, Cohen’s *d* = 0.89; frequency: − 1.19 [− 1.87; − 0.51], *p* = 0.006, Cohen’s *d* = 0.55).

**Table 2:** Change from baseline to week 4 in hot flush symptoms and quality of life.

Outcomes, metric	Baseline (observed)				Mean change from baseline to treatment end (week 4) (estimates)				
	OLP (*N* = 50)		No-treatment (*N* = 50)		OLP	No-treatment	Group difference [95% CI]	*p*	Cohen’s *d*
	*M*	*SD*	*M*	*SD*	*M*	*M*			
Log-transformed hot flush score (diary)^a^	2.75	0.49	2.85	0.51	− 0.52	− 0.20	− 0.32 [− 0.43; − 0.21]	< 0.001***	0.86
Problem rating (HFRS)^b^	6.45	1.24	7.12	1.20	− 1.86	− 1.43	− 0.43 [− 1.01; 0.15]	0.24	0.23
Hot flush frequency (Diary)	8.58	3.90	8.95	3.73	− 2.90	− 1.77	− 1.12 [− 1.81; − 0.43]	0.02*	0.51
QoL overall (MRS-II)^c^	18.06	9.25	18.86	7.87	− 1.75	0.78	− 2.53 [− 4.17; − 0.89]	0.02*	0.49
QoL Anxiety and Depression (WHQ)^d^	75.67	21.59	75.43	16.22	2.22	0.86	1.37 [− 1.82; 4.56]	0.50	0.14
QoL Well-being (WHQ)	80.78	16.48	80.33	14.16	− 2.01	− 1.00	− 1.01 [− 4.73; 2.71]	0.67	0.09
QoL Somatic symptoms (WHQ)	69.47	20.39	69.60	15.60	3.18	− 1.73	4.92 [0.05; 9.98]	0.05	0.39
QoL Memory/concentration (WHQ)	55.56	29.78	50.67	24.81	4.70	1.78	2.92 [− 1.63; 7.49]	0.31	0.20
QoL Sleep problems (WHQ)	42.33	26.77	43.33	30.12	5.38	1.33	4.05 [− 2.00; 10.09]	0.29	0.20

Baseline values were obtained from the observed means and standard deviation. The mean change from baseline to week 4 was obtained from estimates of linear mixed models.

*OLP *Open-label placebo, *HFRS *Hot Flush Rating Scale, *QoL *Quality-of-Life, *MRS-II *Menopause Rating Scale II, *WHQ *Women’s Health Questionnaire, *PGIC *Patient Global Impression of Change.

*p < 0.05, ***p < 0.001.

^a^Hot flush score is a composite scale of frequency × severity (1 = mild, 2 = moderate, 3 = severe).

^b^Scale range: 1–10.

^c^Scale range: 0–55. Higher scores indicate severe menopausal symptoms and great impact of such symptoms on quality of life.

^d^Scale range of all WHQ subdomains: 0–100. Higher scores indicate better health.

Problem rating, i.e., the perceived burden by hot flushes, decreased within both groups (OLP: − 1.86, 95% CI [− 2.38; − 1.34], *p* < 0.001; No-treatment: − 1.43 [− 1.94, − 0.91], *p* < 0.001), yet did not differ *between* the groups (− 0.43 [− 1.01; 0.15], *p* = 0.24, Cohen’s *d* = 0.23). We found a significant group difference of moderate size (Cohen’s *d* = 0.51) for overall menopause-related quality of life (MRS-II). However, there were no differences in the menopause-related quality of life subdomains (WHQ). Only for the subdomain somatic symptoms, the groups differed in trend (4.92 [0.05; 9.98], *p* = 0.05, Cohen’s *d* = 0.39). Since overall and specific quality of life scales demonstrated different results, we deleted the “vasomotor symptoms” item from the overall scale for further inspection. We found the same results as with the original scale (− 2.47 [− 3.99; − 0.95], *p* = 0.01, Cohen’s *d* = 0.51).

As some scenarios of the pre-planned sensitivity analyses did not apply to this sample, we omitted three of the four sensitivity analyses. Eleven patients (OLP: 5, no-treatment: 6) made lifestyle changes during the treatment yet removing them did not modify the results (log-transformed hot flush score: − 0.32 [− 0.44; − 0.19], *p* < 0.001, Cohen’s *d* = 0.86). Changes in lifestyle were non-substantial, i.e., women were less physically active or drank less alcohol in comparison to baseline. We skipped re-rerunning the analyses after imputing missing values of dropped cases, as there was only one patient who did not complete assessments at treatment end. This low percentage of missing data is unlikely to create biases in estimates of linear mixed models^31^. We also skipped re-rerunning the analyses after deleting non-adherers since all patients adhered to the treatment. Another sensitivity analysis would include stress and years since onset of hot flushes as covariates given its role as potential confounders. However, in this sample, neither stress (*r* = 0.11, *p* = 0.30), nor years since onset of hot flushes (*r* = − 0.04, *p* = 0.71) correlated with the change of log-transformed hot flush score, rendering further analyses futile. We omitted sensitivity analyses with problem rating, as there was no treatment effect.

### Duration and time course of OLP effects

Within the OLP group, the log-transformed hot flush score did not differ between week 4 and week 8 (− 0.04 [− 0.06; 0.14], *p* = 0.45). This pattern was also found within OLP 4 week (n = 21), for which benefits remained at week 8 (− 0.03 [− 0.11; 0.17], *p* = 0.69). Correspondingly, problem rating did not differ between week 4 and week 8, neither in the OLP group (− 0.001 [− 0.46; 0.46], *p* = 0.99), nor in the OLP 4 week subgroup (0.05 [− 0.59; 0.69], *p* = 0.89). Furthermore, there were no differences between the OLP 8 week and OLP 4 week at week 8 (log-transformed hot flush score: 0.04 [− 0.17; 0.25], *p* = 0.73; problem rating: − 0.07 [− 1.17; 1.08], *p* = 0.90). Taken together, these results indicate that benefits remained after intake was stopped. Taking the placebo for 8 weeks was not superior to taking the placebo for 4 weeks. Measures of distribution of all outcomes in the follow-up period are listed in the Supplementary Table S1.

**Figure 3:**
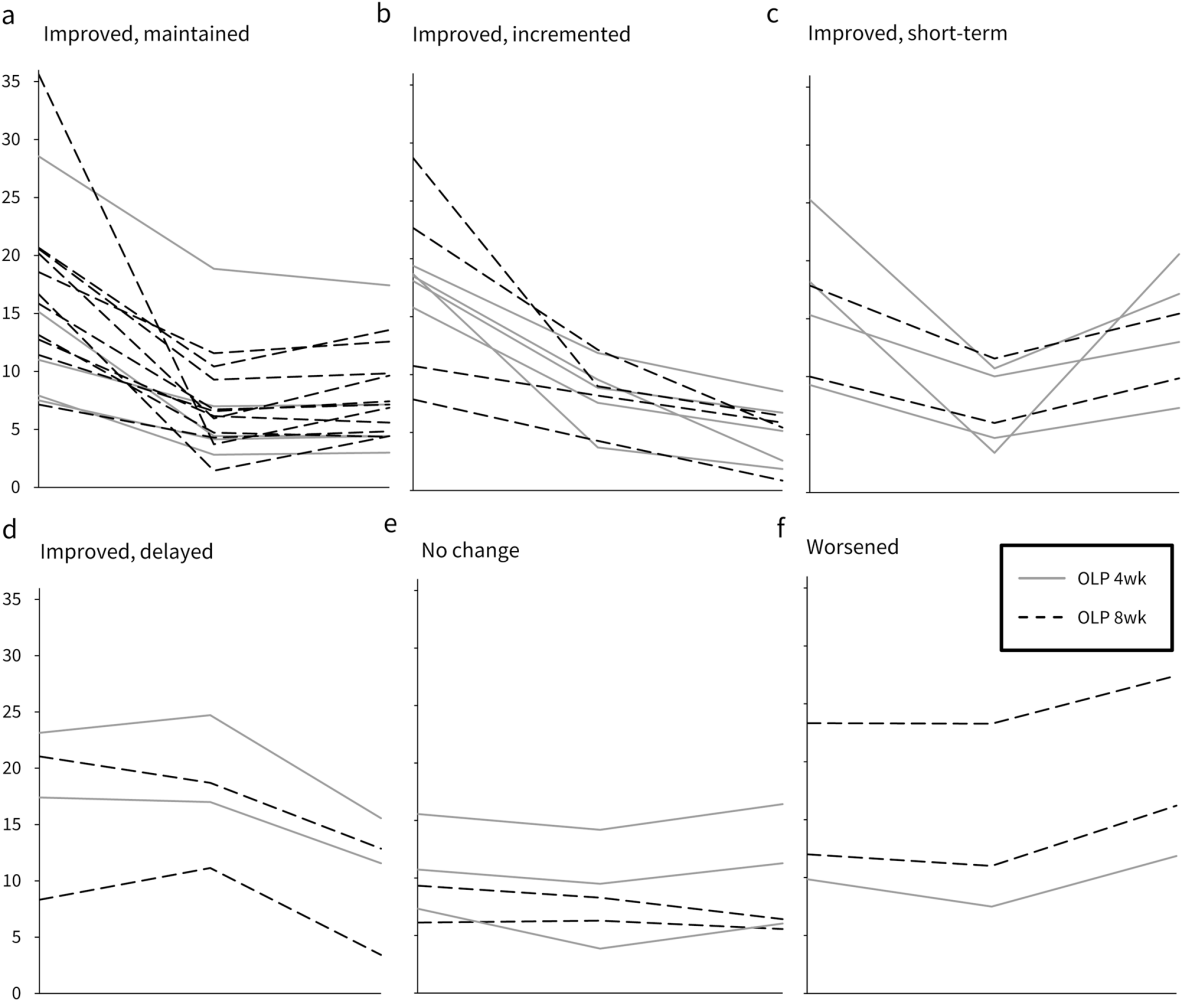
Individual trajectories of hot flush score from baseline, week 4, to week 8 (observed values). Each line represents one case. *OLP 4wk *OLP intake from baseline to week 4, *OLP 8wk *OLP intake from baseline to week 8.

When examining the individual trajectories of the hot flush score over time, we found six different patterns (Fig. 3): Improved, maintained (37%), Improved, incremented (21%), Improved, short-term (14%), Improved, delayed (9%), No change (12%), and Worsened (7%). Descriptive statistics displayed no distinct group differences considering the symptom course. In particular, of OLP 8 week and OLP 4 week, 16 (73%) and 13 (62%) women reported maintained, incremented, or delayed improvements. Summarized, visualizations of the symptom course validated the statistical analyses.

### Role of expectation, hope, and optimism

On average, hope was higher than expectations at all time points (Table 3). Expectations at baseline were right-skewed, i.e., 24% patients expected ‘no change’ after they were randomized. In contrast, hope was left-skewed, i.e., 33% wished for maximum improvement. Table 3 shows that the treatment group predicted expectations after the first randomization (*p* = 0.005), indicating that patients expected more improvement when allocated to OLP. After the second randomization, expectations did not differ between OLP 8 week and OLP 4 week (*p* = 0.48). This indicated that treatment continuation or discontinuation did not affect expecting improvements. Hope was affected by neither of the two randomizations.

**Table 3:** Group differences in expectations of and hope for hot flush improvement.

	Enrolment (M, SD)^a^	Baseline (M, SD)^a^	OLP vs. no-treatment	Week 4 (M, SD)^b^	OLP 8 week vs. OLP 4 week	Week 8 (M, SD)^c^
**Expectations**						
No-treatment	2.28 (2.65)	2.88 (2.62)	β = − 0.24, *p* = 0.005	2.88 (3.02)		
OLP	3.22 (2.84)	4.76 (2.93)		See subgroups		
OLP 8 week				5.18 (3.54)	β = − 0.12, *p* = 0.20	4.64 (3.66)
OLP 4 week				4.43 (3.20)		3.29 (3.44)
**Hope**						
No-treatment	5.70 (3.18)	6.88 (3.09)	β = − 0.11, *p* = 0.48^d^	6.33 (3.21)		
OLP	5.80 (3.35)	7.58 (2.44)		See subgroups		
OLP 8 week				6.95 (2.85)	β = − 0.04, *p* = 0.79^d^	5.86 (3.66)
OLP 4 week				6.86 (2.43)		6.81 (2.58)

Patients were asked whether they expected [hoped] that hot flushes would improve in the ‘next week’ (enrolment) or ‘in the next 4 weeks’ (baseline, week 4, week 8). Both scales range from 0 ‘no improvement’ to 10 ‘maximum improvement’. At baseline and week 4, expectation and hope were assessed *after* the randomization. Tests of group differences were adjusted for the respective value at enrolment.

*OLP *Open-label placebo.

^a^No-treatment: N = 50; OLP: N = 50.

^b^No-treatment: N = 48; OLP 8 week: n = 22; OLP 4 week: n = 21.

^c^OLP 8 week: n = 22; OLP 4 week: n = 21.

^d^Due to violation of homoscedasticity, significance tests were based on standard errors obtained using bootstrapping.

Although the randomization influenced expectations, expectations had no effect on hot flushes. For the complete sample, we found that the baseline to week 4 change in the log-transformed hot flush score did not correlate with expectations at baseline (*r* = − 0.17, *p* = 0.09), nor with hope (*r* = − 0.03, *p* = 0.78), or optimism (*r* = − 0.06, *p* = 0.55) at baseline. We found the same results when correlating expectations with both the non-transformed hot flush score and frequency at week 4. Among these variables, hope correlated significantly with expectations (*r* = 0.39, *p* < 0.001) and optimism (*r* = 0.25, *p* < 0.01). No associations were found between expectations and optimism (*r* = 0.09, *p* = 0.40). As indicated by fixed effects of linear mixed models, there was no main effect of expectations on the log-transformed hot flush score (*F*(1, 94) = 2.30, *p* = 0.13). Also, there was no interaction effect of Expectations x Group x Time on the log-transformed hot flush score (*F*(8, 381) = 0.97, *p* = 0.46). Correspondingly, there was no main effect (*F*(1, 94) = 1.11, *p* = 0.30), or interaction effect (*F*(8, 380) = 1.01, *p* = 0.43) of hope. We found the same pattern for optimism (Main: *F*(1, 94) = 0.59, *p* = 0.45; Interaction: *F*(8, 381) = 0.68, *p* = 0.71). As there was no treatment effect of problem rating, analyses to assess potential moderation effects by expectations, hope, and optimism were omitted.

### Adherence

At week 4, 33 (66%) and seventeen patients (34%) reported having taken ‘all’ and ‘almost all’ pills, respectively. No patients took less than ‘almost all’ pills. Therefore, all patients were adherent according to our criterion. Of those who took ‘almost all’ pills, six additionally specified that they had forgotten one or two pills. Others did not give further information. At week 8, eighteen (82%) and four patients (18%) indicated to have taken ‘all’ and ‘almost all’ pills, respectively. Adherence at week 8 was reported by the OLP 8 week group only.

### Adverse events

We examined whether there were placebo-related adverse events. As shown in Table 4, the no-treatment group reported *more* symptoms at treatment end than the OLP group, indicating that these symptoms may be everyday life complaints. It should be mentioned that some of these symptoms—which are frequent adverse events—might also be related to menopause, including sleeping problems, fatigue, nervousness, and mood swings.

**Table 4:** Baseline-adjusted occurrence of adverse events.

	Open-label placebo (n = 50)	No-treatment (N = 50)
Decreased appetite	3	3
Dry mouth	5	9
Sleeping problems	2	5
Nausea	2	9
Dizziness	7	7
Fatigue	5	12
Constipation	6	3
Nervousness	6	8
Mood swings	6	10

Six and three patients in the OLP and the no-treatment group respectively reported that their hot flushes had gotten worse (“much worse” or “minimally worse”). One patient in the no-treatment group got “very much worse”. Three cases dropped out due to symptom aggravation, of which one (no-treatment group) dropped out during the treatment phase, and two (OLP) dropped out after the treatment phase, i.e., did not participate in the follow-up. One patient refused to continue the treatment for another 4 weeks after increased rates of diarrhoea during the first 4 weeks of treatment. Abdominal symptoms are unlikely to be caused by the pills’ lactose loads^32^ yet may be associated with nocebo effects.

### Opinion of OLP

Patients’ opinions were assessed at week 4, i.e., after the treatment phase. Of 97 patients who stated an opinion, 70% thought that OLP usage in clinical practice is ‘definitely legitimate’. Another 25% indicated ‘rather yes’ whereas 8% indicated ‘rather no’. Some women specified their choice. Quotes are shown in Supplementary Table S2.

## Discussion

In this first study to investigate open-label placebos for menopausal hot flushes, we found that hot flush symptoms decreased significantly in comparison to no-treatment. The primary outcome hot flush score decreased by 43% after 4 weeks of placebo intake, which was significantly better than a 19% reduction in the no-treatment group. We also found a decrease in daily hot flushes by 33% and improvements in overall menopause-related quality-of-life. Overall improvement was reported by 62% of women in the OLP group, whereas a clinically meaningful improvement, i.e., 50% lesser hot flushes, was obtained by 24%. In comparison, many women in the no-treatment also reported overall improvement (40%), yet only 6% achieved hot flush reductions that were clinically meaningful. Compared with control, OLP reduced the number of hot flushes per day by 1.12 points. This change is similar to reductions achieved with SSRI/SNRI (1.13), soy isoflavones (1.15), and clonidine (0.95)^33^, yet minor in relation to hormonal therapy (16.8–22.4, depending on the active ingredient)^26,33^.

The treatment effect on the hot flush score, which we log-transformed in the analyses, was large. The magnitude of this effect aligns with previous OLP studies of irritable bowel syndrome^3^ or chronic low back pain^4^. It is well established that placebo effects in double-blind hot flush trials are substantial, with rates ranging between 20 and 50%^20^. Our study demonstrated that openly administered placebos show benefits similar to placebos given under double-blind uncertainty.

We found no treatment effect on problem rating. Problem rating refers to the degree to which hot flushes are regarded as problematic, distressing, and causing interference with day-to-day life. It is argued that problem rating, not numbers or severity of hot flushes per se, determines whether a patient would seek treatment^11^. This outcome was mainly included in psychological interventions for hot flushes. Improvements in problem rating were achieved by cognitive-behavioural therapy (CBT)^24,34^ but not by mindfulness-based stress reduction (MBSR)^35^. During CBT, patients learn to better manage their hot flushes, e.g., in social situations in which women often feel embarrassed when flushed or considering sleeping problems^34^. Similar to MBSR, our treatment did not directly target hot flush management, which presumably explains the lack of a treatment effect. However, problem ratings were significantly reduced within both groups. In particular, problem rating decreased by 1.9 points in the OLP group, and by 1.4 points in the no-treatment group. The benefits in the OLP group are noteworthy, given that a 2-point reduction is considered clinically significant^34^. We hypothesize these improvements to be related to the three study visits in which patients interacted with a warm, empathetic clinician. A positive patient-clinician relationship is known to be beneficial for health outcomes^36,37^ and is discussed to have also caused improvements in the no-treatment arm of previous OLP studies^3^.

There was a positive effect on the overall quality of life, but not on the subdomains of quality of life. These included anxiety and depression, well-being, somatic symptoms, memory/concentration, and sleep problems. A reduction in hot flushes might have brought about collateral benefits to other complaints like insomnia, fatigue, irritability, and depressed mood-symptoms that were recorded with the overall quality of life scale. However, these secondary benefits were seemingly not large enough to cause significant reductions in each subdomain. These findings are comparable to the first OLP RCT with irritable bowel syndrome patients in which the groups differed in trend considering quality of life^3^.

Notably, the benefits of the placebo persisted beyond intake. On average, and for most patients (52%) who discontinued after 4 weeks, improvements remained for another 4 weeks. Interestingly, taking the placebo for 4 or 8 weeks did not affect whether the benefits remained at follow-up. Freeman has reported comparable findings for double-blind drug trials of hot flushes^25^. After discontinuing or tapering placebo pills, most patients remained improved. Sustained OLP effects were also found in the first two studies including exploratory follow-up phases with cancer patients with chronic fatigue after undergoing chemotherapy^8^ and chronic back pain patients^5^.

There is an ongoing debate about the underlying mechanisms of open-label placebos. Expectations are—alongside with conditioning—the most researched and established mechanisms of placebo effects in general^38^. Researchers have argued that, by providing all patients with a briefing about the placebo effect at the beginning, i.e. prior to randomization, positive expectations might have been elicited^39^. Expecting treatment benefits in turn might influence actual treatment outcomes. Indeed, after randomization, the OLP group expected more improvements than the no-treatment group. However, there was no link between expectations and change in hot flush scores. These results align with a previous study on acupuncture for hot flushes, in which expectations were also not predictive of symptom change^40^. After taking 4 weeks of placebo, patients were randomized again; this time to either continue or discontinue the OLP treatment. Although descriptively, expectations were higher when treatment continued, the difference between the groups was nonetheless smaller in comparison to the first allocation. Whereas patients had to rely on “external” information when formulating their expectations at the first allocation, they were able to incorporate first-hand experiences in the second allocation. Overall, our results indicate that the relationship between expectations and treatment benefits is not as straightforward, i.e., simply expecting to get better is not the reason why someone does get better. However, we should note that our design is not suited or aimed at testing underlying mechanisms of OLP effects. This is better investigated in studies in which OLP is paired with different kinds of information that aim at eliciting different degrees of expectations^41,42^. Aside from expectation, we also looked at hope for improvement and optimism. Hope was not affected by the group assignment and was higher than expectations, which aligns with qualitative studies on complementary and alternative therapies^43,44^.

Like expectations, hope also did not predict change in hot flush score or moderate the treatment effect. Moreover, we found no link between optimism and the outcome, which aligns with a previous study that reported no predictive value of optimism on OLP effects in healthy participants^45^. Some researchers have begun to propose that OLP relies on non-conscious Bayesian cortical processing that can significantly shape perceptions of symptoms^46^. Taken together, understanding the underlying mechanisms of OLP requires further investigation.

Some limitations should be noted. First, our follow-up period was rather short. The 4-week follow-up allows for first insights considering the time course of placebo effects yet is too short to evaluate clinical usefulness. The European Medicines Agency, for example, requires a follow-up period of 12-week for market approval of hot flush remedies^47^. Second, we recruited via the general population. Therefore, symptoms were not validated by gynaecologists. However, our objective was to reach those women who suffered from hot flushes yet did not see a practitioner since they rejected hormone therapy^48^. As reflected in our baseline characteristics, most patients have used remedies like for example black cohosh, homoeopathic remedies, sage, etc. which are sold as over-the-counter medicines or food supplements and do not require seeing a practitioner. Considering that practitioners also mostly rely on women’s self-reports—hormonal tests for women over 45 with irregular or absent menses to determine menopausal status are explicitly discouraged in the guidelines^21,22^—we believe our procedure to be valid. Besides, we excluded other potential causes of flushing via the structured screening interview. The third limitation concerns adherence. First of all, we assessed adherence only through self-report. Secondly, we defined adherence as taking more than 80% of all pills, which applied to all patients. However, 34% indicated to have taken “almost all” pills. Based on the participants’ feedback during the study visits, we presume that this patient sample is not used to taking medications daily, thus making it likely for women to forget one or two pills. Other frequent reasons for non-adherence such as complexity of regimen and side effect burden do not apply to our treatment^49^. We also aimed to establish a supportive patient-clinician relationship based on trust, which in turn can enhance adherence. However, since forgetfulness is one of the most common reasons for non-adherence^49^, future studies might consider using medication-related strategies to increase adherence, like providing pill boxes or notifications via an App. Safeguarding adherence is especially relevant for future OLP studies with treatment periods longer than three or four weeks. Lastly, in our sample, the total symptom duration ranged between 30 days and 26 years, thus constituting both a potential limitation and a strength with regards to ecological validity.

Does this study warrant the use of OLP in clinical practice? Since this is the first study to investigate OLP for hot flushes with a short follow-up period and a rather small sample size in comparison to trials of other hot flush remedies, we believe that further evidence is needed to draw definite recommendations. However, our findings have clinical value as they provide a basis for re-evaluating the role of placebo treatments in medical practice. Given that complementary and alternative treatments do not outperform placebos in RCTs but are nonetheless commonly used by menopausal women^48,50^, it may be conceivable to consider OLP as an additional option. In the German S3-clinical guidelines of treating peri- and postmenopausal symptoms, placebos are already placed as an optional hot flush treatment next to, among others, phytoestrogen and CBT^21^. Therefore, in Germany, specifically, our study provides a basis for clinicians to administer placebos under ethical values. It is important to note that we tested the efficacy of OLP within a clinical context. That is, we are uncertain about whether women self-administering placebo treatment would be as effective as prescribing placebo by a health care provider, given that evidence strongly suggests that placebo effects are enhanced by a supportive relationship^36,37,51^ In summary, the positive effects of OLP in this trial indicate a potential for OLP to be a safe, effective treatment for women with moderate and severe hot flushes.

## Methods

### Trial design

The study protocol^52^ provides a detailed description of the design. Briefly, in this parallel group, two-arm, superiority trial, participants were allocated 1:1 to receive 4 weeks of open-label placebos or no treatment. During the study period, participants completed a hot flush diary. A phone interview that was conducted by study assistants screened for eligibility. We formed a stringent questionnaire that either qualified or disqualified women for study participation. Enrolled women received three study visits in which they interacted with a clinician and completed questionnaires. The OLP group was randomized a second time at the third study visit to either take another four weeks of open-label placebos (OLP 8 week) or to discontinue their OLP treatment (OLP 4 week). To obtain a baseline on hot flushes, women completed the diary for 7 days before randomization, i.e., between study visit 1 and 2. The first randomization took place at study visit 2. In turn, study visits 3, and 4 (OLP 8 week and OLP 4 week only) took place at 4 and 8 weeks after the first randomization. The clinician conducted short calls inquiring for symptom status, potential adverse effects, and adherence (OLP only) at 2 and 6 weeks after the first randomization. Ethical approval was given by the ethics committee of the Hamburg Medical Association (“Hamburger Ärztekammer”) (trial number: PV5787), and experiments were performed in accordance with the Declaration of Helsinki^53^. The trial was registered on 12/02/2019 at ClinicalTrials.gov (registration number: NCT03838523). Written informed consent was given by all participants prior to enrolment. This trial was conducted at the University Medical Centre Hamburg-Eppendorf, Department of Psychosomatic Medicine.

### Participants

We included women who were post-menopausal (12 months from last menstruation), or in menopausal transition (amenorrhea ≥ 60 days in the past year). Eligibility criteria required women to have at least five daily hot flushes of moderate or severe intensity which were considered problematic (16+ on the problem rating scale of the Hot Flush Rating Scale^54^). Women who used any kind of hot flush treatment or SSRI/SSNI within the past 6 weeks were excluded. Also, other potential causes of flushes were excluded: risk of an alcohol use disorder (5+ on the Alcohol Consumption Questions^55^), cancer diagnosis within the past ten years, current endocrine therapy for breast cancer, untreated hyperthyroidism, high levels of either depression, or anxiety, or both (9+ on the Patient Health Questionnaire-4^56^ or 5+ on one of its subscales). Mind–body interventions like yoga, meditation, etc., were permitted if they were ongoing throughout the treatment. At informed consent, all patients were encouraged to refrain from lifestyle changes during their participation in the trial.

### Interventions

Both groups underwent the same interactions with the clinician (female psychologist) and received the same information about open-label placebos. The no-treatment group controls for spontaneous improvement, regression to the mean, and normal fluctuations in symptoms. The clinician pursued to treat all patients in an empathetic, warm manner.

The clinician informed all participants that the placebo contained no active ingredient and was made of lactose. At study visit 1, i.e., prior randomization, the clinician takes a history and inquiries about relevant biopsychosocial information (10–15 min). Afterwards, each patient receives the following information about placebos^3^: (1) the placebo effect is powerful; patients reported symptom improvement after taking placebos in double-blind drug trials, including hot flush trials. However, these participants were unaware of whether they were receiving a placebo or medicine. (2) A few studies have shown that placebos without deception can have beneficial effects. The body may react to the pill intake automatically (Depending on the patient’s prior knowledge, an example is given, e.g., the Pavlov dog or food poisoning for which certain foods cause queasiness and nausea). Being positive and believing in a positive effect can help but is not necessary. (4) Taking the pills faithfully twice a day is crucial since the custom of pill intake can contribute to the effect. Also, adhering to the instructions is important to maintain the quality of the study, i.e., if some patients would only take half their pills, we would be comparing several subgroups with the no-treatment group. (5) Finally, we do not know whether placebos without deception can reduce hot flushes. Therefore, we encourage patients to simply “give it a try.”

The groups differ solely considering pill administration plus intake and inquiries about adherence. At study visit 2, patients were randomized. Patients in the OLP group received a glass bottle containing the pills labelled “Placebos for menopausal hot flushes” with the original medication leaflet. The placebo pills (white and uncoated) were manufactured by the company “Zentiva Pharma GmbH” and bottled by the UMC Hamburg pharmacy for this study. The no-treatment group was reminded about the importance of the control group and to complete the diary faithfully. The no-treatment group received placebo pills at the end of the study if desired.

### Outcomes

Given the novelty of this study, we included two primary outcomes: group difference in hot flush score change from baseline to treatment end (week 4), and group difference in problem rating change measured with the Hot Flush Rating Scale (HFRS^54^). The hot flush score (frequency × severity), measured with a diary, is the most often used outcome in hot flush trials^57^. The diary is considered a gold standard to assess hot flushes^58^. Flushes during daytime were recorded upon occurrence, and night sweats were recorded upon awakening. To facilitate recording, patients received separate diaries for day and night. Categories of each hot flush (1 = mild, 2 = moderate, 3 = severe) were pre-defined following the European Medicine Agency’s guidelines^47^. Problem rating refers to the perceived hot flush burden considering cognition, emotion, and day-to-day interference. A 2-point improvement is regarded as clinically relevant^34^. Both the diary and the HFRS have demonstrated good reliability and validity^54,58^.

Secondary outcomes include change in hot flush frequency, change in health-related quality of life, overall improvement (PGIC)^59^, and number of responders (50% reduction in frequency from baseline to treatment end). Frequency is defined as the number of hot flushes per 24 h. The overall quality of life was measured with the Menopause Rating Scale-II (MRS-II)^60,61^, whereas subdomains of quality of life were measured with the Women’s Health Questionnaire (WHQ-23)^62^. The MRS-II evaluates the severity of 11 common menopausal complaints. Of the original eight WHQ scales, we excluded the optional scales ‘menstrual symptoms’ and ‘sexual behaviour’ due to low relevance in this sample (latter scale completed by n = 40 women). The ‘vasomotor symptoms’ scale was omitted due to redundancy. Analyses were conducted with the subdomains ‘anxiety and depression’, ‘well-being’ [“feels well with herself (good appetite, feels physically attractive)”], ‘somatic symptoms’, ‘memory/concentration’, and ‘sleep problems’. Each subdomain ranges from 1 to 100, with higher scores indicating better health. Improvements of 10–20 points are considered clinically relevant^63^. For both quality of life questionnaires, validity, good reliability and sensitivity to change have been demonstrated^60,62^. PGIC assessed the subjective overall improvement of hot flushes within the past four weeks from 1 ‘very much worse’ to 7 ‘very much better’^59^. We calculated a binary scale for easier interpretation, with scores larger than 4 (‘no change’) being categorized as ‘improved’. To obtain the number of responders, we compared the hot flush frequency at baseline with the frequency at treatment end.

### Further assessments

We assessed expectations and hope of symptom improvement at each study visit on a scale from 0 ‘no change’ to 10 ‘maximum change’. As there was no validated questionnaire when the study was designed, we created one-item scales based on interview questions in a relevant qualitative study^64^. At baseline, we additionally measured stress (Perceived Stress Scale-10^65^), and dispositional optimism (Life orientation test-revised^66^). At treatment end, patients indicated whether they made any lifestyle changes considering smoking, alcohol, exercise, diet, and mind–body treatments. Patients also rated whether OLP use in clinical practice is acceptable from 1 ‘definitely yes’ to 4 ‘definitely not’^67^. Adherence is assessed 4 and 8 weeks after randomization using one item (“How many placebo pills have you taken in the last week?”) ranging from 0 ‘none’ to 6 ‘all’^68^. Patients who indicated to have taken ‘almost all’ and ‘all’ their pills were considered adherent. Adverse events (e.g., nocebo effects) were recorded using the Generic Assessment of Side Effects^69^. We chose the nine most frequent symptoms that were reported by women of the German general population, i.e., reduced appetite, dry mouth, sleeping problems, nausea, dizziness, fatigue, constipation, nervousness, and mood swings^69^. Symptoms that were already indicated at baseline were not classified as adverse events.

### Sample size

Previous OLP studies have found large effects of Cohen’s *d* = 0.80^3,4^ and hot flush double-blind trials have reported placebo improvements of moderate size (*d* = 0.40)^70^. Given a presumed effect of *d* = 0.60, an alpha of 0.05 (two-tailed), 80% power, and finally, an attrition rate of 10%, we obtained our required sample of *N* = 100. The sample size was calculated with G-Power.

### Randomization

The clinician conducted the randomization at study visit 2 by opening a sequentially numbered, opaque envelope. The group affiliation was communicated to the patient immediately after the reveal. We used a permuted block randomization with block sizes of 4 and 6.

We conducted a second randomization at study visit 3 among the OLP group, stratified by improvement (yes/no). After inquiring about the stratum, the clinician draws the next envelope from the corresponding deck and reveals the group affiliation to the patient. In alignment with the first randomization, envelopes were sequentially numbered and opaque. We used a stratified permuted block randomization with block sizes of 2 and 4.

Both sequences were generated using an online program (Sealed Envelope). A research assistant who had no interaction with patients generated the sequences and prepared the envelopes. The sequences were locked away before enrolment to avert access by study personnel. Similarly, block sizes remained unknown to the study personnel throughout the study.

### Blinding

Due to the nature of the study, patients and the clinician were not blinded. Assessments of primary and secondary outcomes were conducted by research assistants who were blinded to allocation (assessors). At each study visit, clinician consultation and assessment took place in separate rooms. Both the clinician and the assessor reminded patients of not revealing their group affiliation when completing the questionnaires, verbally or by accident (e.g., putting placebo pills next to the questionnaire after receiving them). Group affiliation in questionnaires was masked by using letters (A vs. B and C vs. D) instead of group names.

### Handling missing values

Provided that there is 80% of data on each scale, missing items were substituted by the mean of the non-missing items^71^. The single-item scales expectations and hope were not replaced. Missing data points in the diary were imputed separately for daytime and night-time. That is, missing data points of night sweats were substituted by the average night sweats of the rest of the week; computation of the 24 h-value followed hereafter. A previous study has found no considerable difference between a 3-day and a 7-day weekly mean of the hot flush diary^72^. Missing values (per scale or data point) were not imputed since linear mixed models allow statistical evaluation of incomplete data^31^. Missing values of categorical variables (if < 5%) were imputed with the most likely answer. That is, in the case of dropout, the categorical outcomes ‘improved (yes/no)’ and ‘responded (yes/no)’ were imputed with ‘no’.

### Statistical analyses

#### Efficacy

For metric variables, we conducted linear mixed models for repeated measures. Measurement points were nested within participants. For hot flush score and frequency, we computed a weekly average per 24 h, thus obtaining five measurement points for each outcome. The hot flush score is a composite score and was right-skewed. Since the values were arbitrary, we performed a log-transformation to obtain distributions closer to normal. Problem rating and quality of life scales were assessed at baseline and treatment end, yielding two measurement points per outcome. We included the fixed effects Time (Factor), Group, Time × Group, and modelled the Intercept as a random effect. For the hot flush score, frequency, problem rating, and overall quality of life (MRS-II), we included the respective grand-mean centred baseline value as a covariate. Models of the quality of life subdomains (WHQ) did not converge when the respective baseline value of each domain was included. Consequently, we included overall quality of life (MRS-II) at baseline as a covariate for all quality of life variables. Estimates were obtained with the restricted maximum likelihood (REML) method. A heterogeneous autoregressive covariance structure type was set as the default. If the model did not converge, we chose an easier model, i.e., proceeded with a more restrictive covariance structure type until conversion was reached (heterogeneous autoregressive to homogenous autoregressive to variance components). Categorical variables, including improvement (yes/no) and responding to treatment (yes/no), were analysed with Chi-Square tests and Fisher’s Exact Test (cell number < 5), respectively.

Sensitivity analyses for primary outcomes were pre-defined in the study protocol and included: (1) imputing missing values of dropped cases, (2) deleting cases who were non-adherent, (3) statistically adjusting for perceived stress and years of hot flushes (potential confounders)^11^, and (4) excluding patients who made lifestyle changes.

#### Expectations, hope, and optimism

We conducted regression analyses to examine whether expectations and hope differed between the groups. All analyses were adjusted for its respective value at enrolment. To investigate whether expectation at baseline predicted or/and moderated the treatment effect, we extended the model of our primary outcome by Expectation, Expectation × Group, and Expectation × Group × Time. Matching analyses were conducted for hope and optimism.

#### Maintenance and duration

We were interested in the following questions: First, do benefits sustain in the OLP group at follow-up end (week 8)? Second, do benefits sustain in the OLP 4 week group at follow-up end? Third, do OLP 8 week and OLP 4 week differ at 8 weeks? Fourth, which kinds of symptom trajectories are present? For questions 1–3, we conducted a linear mixed model to compare the week 4 to week 8 change in log-transformed hot flushes. We included the fixed effects Time (factor with five categories: week 4, 5, 6, 7, 8), Intake Duration (OLP 8 week vs. OLP 4 week), Time × Intake Duration, and the centred log-transformed score at week 4. For question one, we compared the score at week 4 and week 8 within OLP. For question two, we conducted the same comparison within OLP 4 week. For question three, we compared OLP 8 week and OLP 4 week at week 8. Model specifications corresponded with the ones for the efficacy analyses. For question 4, we grouped symptom trajectories based on visual examination and patients’ improvement ratings. All analyses on the follow-up period were pre-defined in the protocol and limited to primary outcomes. Given the small sample size of the OLP subgroups, we prioritize descriptive data over statistical significance when interpreting the data.

#### Assumptions

Prior analyses, we inspected model assumptions of linear modelling: Homoscedasticity, normality of residuals, no multicollinearity or multivariable outliers. To comply with standards of intention-to-treat analyses, we report results with the full sample. In the case of multivariate outliers, results are compared between the analyses, including and excluding the outlier. In case homoscedasticity was not met, we obtained robust errors by using bootstrap methods (1000 samples, bias and accelerated method). Normality of residuals was met for all analyses.

IBM SPSS 26.0 was used for all analyses. We used the MIXED command to conduct linear mixed models and the EMMEANS subcommand for significance testing. Effect sizes of pairwise comparisons of linear mixed models are calculated by dividing the mean group difference by the pooled standard devitation^73^. Tests were two-tailed with an alpha error set at 0.05. Effects of 0.2, 0.5, 0.8 (Cohen’s *d*) and 0.1, 0.3, 0.5 (Cramer’s *V*, *df* = 1) were considered small, moderate, large effects^73^.

## Supplementary information

Supplementary information 1.

Supplementary information 2.

Supplementary information 3.

## Data Availability

The datasets generated during and/or analysed during the current study are available from the corresponding author on reasonable request.
