# Supplementary material for: Open-label placebos for menopausal hot flushes: a randomized controlled trial
Source: Sci Rep. 2020 Nov 18;10:20090. doi: 10.1038/s41598-020-77255-z (PMC7674475; doi:10.1038/s41598-020-77255-z)
Supplement: Supplementary file 2 — Supplementary information 2. [file 41598_2020_77255_MOESM2_ESM.pdf]

Open-label placebos for menopausal hot flushes – a randomized controlled trial

Yiqi Pan[1,2,*], Ramona Meister[3], Bernd Löwe[1], Ted J Kaptchuk[4], Kai J Buhling[5], Yvonne Nestoriuc[1,2]

[1] University Medical Centre Hamburg-Eppendorf, Department of Psychosomatic Medicine and Psychotherapy, Hamburg, 20251, Germany

[2] Helmut Schmidt University / University of the Federal Armed Forces Hamburg, Department of Psychology, Hamburg, 22043, Germany

[3] University Medical Centre Hamburg-Eppendorf, Department of Medical Psychology, Hamburg, 20251, Germany

[4] Harvard Medical School, Beth Israel Deaconess Medical Center, Program in Placebo Studies and the Therapeutic Encounter (PiPS), Boston, 02215, USA

[5] University Medical Centre Hamburg-Eppendorf, Clinic of Gynecology, Department of Gynecological Endocrinology, Hamburg, 20251, Germany

*Corresponding author: y.pan.ext@uke.de

Supplementary Table S1. Descriptive Hot Flush Symptoms, Quality-of-Life, and Overall Improvement over Time by Placebo Intake Duration (8 weeks vs. 4 weeks)

	OLP 8wk (n = 22)						OLP 4wk (n = 21)					
	Baseline		Treatment end (week 4)		Follow-up end (week 8)		Baseline		Treatment end (week 4)		Follow-up end (week 8)	
Outcomes, metric	M	SD	M	SD	M	SD	M	SD	M	SD	M	SD
Hot flush score (Diary)[1]	15.82	7.43	8.57	4.94	8.94	5.76	15.97	6.02	9.36	5.70	9.40	5.57
Problem rating (HFRS)2	6.50	0.96	4.45	1.92	4.50	2.00	6.32	1.44	4.40	1.85	4.35	2.13
Hot flush frequency (Diary)	7.99	2.83	5.05	2.48	4.93	3.05	8.76	3.28	5.22	2.43	5.30	2.80
QoL Overall (MRS-II)3	17.50	9.69	14.36	8.08	13.90	7.54	17.76	9.28	15.95	8.28	14.57	7.51
QoL Anxiety & Depression (WHQ)[4]	76.52	24.13	80.63	22.10	83.12	19.70	78.46	19.20	80.50	18.98	82.31	17.63
QoL Well-being (WHQ)	82.58	15.41	81.44	12.84	82.20	13.92	80.95	17.71	79.23	18.10	80.95	20.44
QoL Somatic symptoms (WHQ)	69.09	25.49	75.15	21.00	78.48	19.18	72.38	14.34	74.60	13.60	73.97	16.18
QoL Memory/concentration (WHQ)	56.57	31.78	65.15	27.71	64.65	27.78	60.85	27.13	60.32	26.89	64.55	25.97
QoL Sleep problems (WHQ)	50.00	30.43	50.00	31.71	55.30	29.72	38.10	24.23	49.21	29.10	48.41	26.82
Outcomes, categorical			N	%	N	%			N	%	N	%
Improved in the past 4 weeks (PGIC)			13	59.1	13	59.1			16	76.2	11	52.4
Number of responders (50% reduction in hot flush frequency)[5]			5	22.7	6(+1)	27.3			5	23.8	7(+2)	33.3

[1] Hot flush score is a composite scale of frequency x severity (1 = mild, 2 = moderate, 3 = severe).

[2] Scale range: 1 to 10.

[3] Scale range: 0 to 55. Higher scores indicate severe menopausal symptoms and great impact of such symptoms on quality of life.

[4] Scale range of all WHQ domains: 0 to 100. High scores indicate good health status.

[5] At week 8, an additional one and two patients obtained 50% reduction of hot flushes in the OLP 8wk and OLP 4wk groups, respectively.

OLP = Open-label placebo; wk = weeks; HFRS = Hot Flush Rating Scale; QoL = Quality-of-Life; MRS-II = Menopause Rating Scale II, WHQ = Women's Health Questionnaire, PGIC = Patient Global Impression of Change.

Supplementary Table S2. Reasons for and against the use of open-label placebos.

Pro	Contra
"I believe in the body's self-healing powers and the effects of positive thinking." ID 18	"I would be okay with it if placebos were prescribed as part of herbal remedies." ID 66
"If placebos work, they should be favored over pharmacological drugs which most often cause side effects!" ID 91	"If a woman has been taking contraceptives for "decades", maybe the body could be fooled for a while. But in the long run, I rather doubt it." ID 79
"Placebos can contribute to the treatment, and often, being cared for already changes the patient's outlook and feelings. Patients develop a more positive view towards their symptoms or changes in their body." ID 3	"When taking a placebo, one knows that this has no effect which could affect the outcome of the study. There should be further studies in which another group takes placebos without being aware of it." ID 89
"If studies have shown that it has benefits then its usage is definitely warranted." ID 26	
"Placebos make sense and there is scientific proof for it. Important: That it is administered by a doctor." ID 41	
"It worked for me" ID 50	
"It gives you some kind of reassurance, since you're in control more." ID 70	
"If there is a chance of even the slightest improvement, it should be used!" ID 78	
"What I find more questionable is the use of placebos without the patient's knowledge" ID 81	
"I don't see any reasons that speak against it." ID 84	
"Body and mind can be influenced by will power and faith" ID 87	
"I think that the thought that they might help does something to the body and one also adjusts to it [that thought] psychologically." ID 22	
"Gives you hope. I personally have more purposefully observed in what circumstances I have hot flushes." ID 93	
"Justified yes, but perhaps not suited for everybody." ID 98	

Note. Answers to the question "In your opinion, is the use of open-label placebos legitimate in clinical practice? Please specify your choice." Quotes were pre-selected to depict a wide range of opinions. Only 8% were "rather against" the use of open-label placebos.
