# Supplementary material for: Open-label placebos for menopausal hot flushes: a randomized controlled trial
Source: Sci Rep. 2020 Nov 18;10:20090. doi: 10.1038/s41598-020-77255-z (PMC7674475; doi:10.1038/s41598-020-77255-z)
Supplement: Supplementary file 3 — Supplementary information 3. [file 41598_2020_77255_MOESM3_ESM.pdf]

Open-label placebos for menopausal hot flushes – a randomized controlled trial

Yiqi Pan[1,2,*], Ramona Meister[3], Bernd Löwe[1], Ted J Kaptchuk[4], Kai J Buhling[5], Yvonne Nestoriuc[1,2]

[1] University Medical Centre Hamburg-Eppendorf, Department of Psychosomatic Medicine and Psychotherapy, Hamburg, 20251, Germany

[2] Helmut Schmidt University / University of the Federal Armed Forces Hamburg, Department of Psychology, Hamburg, 22043, Germany

[3] University Medical Centre Hamburg-Eppendorf, Department of Medical Psychology, Hamburg, 20251, Germany

[4] Harvard Medical School, Beth Israel Deaconess Medical Center, Program in Placebo Studies and the Therapeutic Encounter (PiPS), Boston, 02215, USA

[5] University Medical Centre Hamburg-Eppendorf, Clinic of Gynecology, Department of Gynecological Endocrinology, Hamburg, 20251, Germany

*Corresponding author: y.pan.ext@uke.de

Trial protocol for open-label placebos for menopausal hot flushes

PROJECT SUMMARY

Background. Beneficial effects of placebos are high in double-blind hot flush trials. Studies in various conditions suggest that honestly prescribed placebos may elicit symptom improvement.

Objective. To determine whether open label placebo (OLP) treatment is efficacious in alleviating hot flushes among peri- and postmenopausal women.

Methods. In this assessor-blinded, randomized-controlled trial, n = 100 women with ≥5 daily hot flushes of at least moderate severity and bothersomeness are assigned 1:1 to a 4-week OLP treatment or no treatment. To explore the duration and maintenance of placebo effects, the OLP group is randomized a second time to either discontinue or continue the OLP treatment for another 4 weeks. All participants receive a briefing about placebo effects and study visits at baseline, post-treatment (4 weeks) and follow-up (8 weeks, OLP group only). Qualitative interviews about subjective experiences with the OLP treatment are conducted.

Outcome measures. Primary outcomes are differences between the OLP and the no-treatment group in the hot flush composite score (frequency x severity) and bother of hot flushes as assessed with the Hot Flush Rating Scale at post-treatment. Secondary outcomes include hot flush frequency, health-related quality of life, global improvement, and the number of responders at post-treatment. Data is analyzed by fitting (generalized) linear mixed models. An exploratory analysis of maintenance and duration is performed including follow-up data.

Discussion. This trial will contribute to the evaluation of OLP treatments in clinical practice and further our understanding about the magnitude of placebo effects in hot flush treatments.

GENERAL INFORMATION

Trial registration: The trial is registered at clinicaltrials.gov (ID: NCT0383852) on 29/01/2019; link: https://clinicaltrials.gov/ct2/show/NCT03838523.

Ethics approval and consent to participate: Ethics approval to the trial protocol version 1.0 (Hamburg, May 22, 2018) has been given by the ethics committee of the Hamburg Medical Association (reference number PV5787). Any amendments to the protocol will be communicated and re-approved by the ethics committee. All participants will be informed about the study in verbal and written form before the consent procedure. Informed consent is obtained from all participants prior enrolment.

Principle investigator: Prof. Yvonne Nestoriuc, Helmut Schmidt University / University of the Federal Armed Forces Hamburg, Department of Psychology, Hamburg, 22043, Germany

Research site (single-site study): University-Medical Center Hamburg-Eppendorf, 20251 Hamburg

Competing interests: The authors declare no conflict of interests.

Funding: The study is conducted with board funds from Yvonne Nestoriuc's lab. This work is additionally supported by the Foundation of the Science for the Therapeutic Encounter, 524 Franklin Street, Cambridge, MA 02139, U.S.A. The funding source had no role in the design of this study and will not have any role during its execution, analyses, interpretation of the data, or decision to submit results.

The protocol has also been published and can be found here:
https://trialsjournal.biomedcentral.com/articles/10.1186/s13063-019-3575-1

BACKGROUND

Hot flushes are the most common symptoms related to menopause (1). Around 16-74% of women worldwide experience hot flushes, persisting on average for 7.4 years, with some women reporting sustained symptoms for up to 14 years (2). Given that about 60% of menopausal women seek treatment (3), improved management of hot flushes is increasingly becoming an important public health issue (4).

The currently recommended (5) and also the most effective treatment is hormone therapy (6, 7). For decades, it was the treatment of choice until the Women's Health Initiative (WHI) study linked hormone therapy to higher risks of developing breast cancer, coronary heart disease, stroke, and endometrial cancer (8, 9). Although the sweeping interpretation has been updated with refined recommendations dependent on individual risk factors, the WHI study nonetheless has been followed by a 79% drop in usage from 2002 to 2010 (10). The challenge has since been to find non-hormonal alternatives in case of refusal or contraindication. Beneficial effects have been shown for selective serotonin reuptake inhibitors, gabapentin, and clonidine (11, 12). Herbal remedies are often used (45-63%) (13-15) although current evidence is mixed with the overall trial quality rated to be suboptimal (16-19). Acupuncture and relaxation techniques were found to be non-effective (20, 21), while cognitive-behavioral therapy, clinical hypnosis, and mindfulness-based stress reduction showed some positive results in alleviating hot flush-related distress (22-24).

Notably, placebo responses are high throughout hot flush trials (25, 26). In studies of non-hormonal therapies, response rates range from 27% to 52% (27). In estrogen trials, participants in the placebo arms obtain an averaged 58% reduction in hot flushes (6). It is unclear whether these beneficial effects reflect a true placebo effect, i.e., a distinctive effect over and above natural history and regression to the mean and if so - whether these effects can be exploited to improve hot flush treatments.

It has been long viewed that placebos cannot be administered in accordance with ethical values since deception would be necessary for the occurrence of beneficial effects. This notion has been shaken up by studies on open-label placebos (OLP), showing that placebos can lead to positive effects even though their inert nature *is* disclosed (28-35). The first randomized-controlled OLP trial demonstrated a significant improvement of irritable bowel syndrome after 3 weeks of placebo intake over an untreated control group with 60 % (vs. 35%) reporting adequate relief (30). Clinical trials have been since conducted among patients with chronic low back pain (28), cancer-related fatigue (36), as well as in smaller trials on depression (31) and allergic rhinitis (32) (33), with reporting of medium to large effect sizes. Mixed results were found in experimental studies with healthy cohorts. Pain (37) and migraine (29) improved after OLP intake, yet wound healing in healthy normal did not (35). Up to now, the number of trials is small and underlying mechanisms are unclear.

The objective of this study is to determine whether OLP is efficacious to treat hot flushes. Further, we will analyze the response rate of OLP and health-related quality of life, whether beneficial effects can be maintained or increased with a prolonged placebo treatment, and whether beneficial effects are related to positive expectations.

METHODS

Study design

In this randomized-controlled, assessor-blinded superiority trial, menopausal women with hot flushes are assigned 1:1 to a 4-week OLP treatment or a no-treatment control group. The control group allows for examining whether OLP has beneficial effects over and above spontaneous improvement and statistical phenomenon. Patients receive three study visits at enrolment, baseline, and post-treatment (Figure 1). Women allocated to OLP are randomized a second time at post-treatment to either another 4 weeks of placebo (OLP 8wk) or to discontinue the treatment (OLP 4wk). Accordingly, OLP 8wk and OLP 4wk receive a fourth study visit at 8 weeks follow-up, whereas, for the no-treatment group, the study ends at week 4. Two calls at week 2 and 6 are conducted to inquire about adverse events and adherence. To further understand the experiences of participants and potential psychological mechanisms from their perspective, qualitative interviews will be conducted with a subgroup of patients who have reported improvements under the placebo. Ethical approval is given by the ethics committee of the Medical Association Hamburg (reference number PV5787). The trial is registered at clinicaltrials.gov (ID: NCT03838523) and complies with the SPIRIT guidelines (38).

		Study period					
Timepoint (weeks)	Scree-ning	Enrol-ment	Base-line*	2wk call	Post	6wk call	8wk FU†
	Prior -1	-1	0	2	4	6	8
**Screening and enrolment**							
Eligibility screen	✓						
Informed consent		✓					
Study visit		✓	✓		✓		✓
Clinical interview		✓					
Briefing: OLP effects and importance of the control group		✓					
Randomized assignment to OLP or no-treatment			✓				
Randomized assignment to OLP 8wk or OLP 4wk‡					✓		
**Intervention**							
No treatment			|----------------------|				
OLP			|----------------------|				
OLP 8wk			|--|				
OLP 4wk			|--|				
**Assessments**							
Hot flush score (frequency x severity) (Hot flush diary)		|--|					
Bother of hot flushes (HFRS)	✓	✓			✓		✓
Hot flush frequency (Hot flush diary)		|--|					
Health-related quality of life (WHQ)			✓		✓		✓
Global Improvement (PGIC)			✓		✓		✓
Alcohol use (AUDIT-C)	✓	✓			✓		✓
Smoking, exercise, diet (varied after MHQ)		✓			✓		✓
Perceived stress (PSS-10)		✓					
Demographic and medical characteristics		✓					
Menopausal symptoms (MRS-II)		✓			✓		✓
Value and probability expectations		✓	✓		✓		✓
Optimism (LOT-R)			✓				
Opinion about OLP usage in clinical practice					✓		✓
Baseline symptoms (GASE)		✓					
Adverse events (open and with GASE)				✓	✓	✓	✓
Adherence and interruption				✓	✓	✓	✓
Perceived improvement (yes/no)					✓		✓
Depression and Anxiety (PHQ-4)	✓						
**Qualitative part**							
Qualitative interview (subgroup of n = 8) §							✓

Figure 1. Schedule of enrolment, intervention and assessment according to SPIRIT. *Post* post-treatment, *wk* week, *FU* follow-up, *OLP* open-label placebo, *HFRS* Hot Flush Rating Scale, *WHQ* Women's Health Questionnaire, *PGIC* Patient Global Impression of Change, *MRS* Menopause Rating Scale, *MHQ* Menopause Health Questionnaire, *AUDIT-C* Alcohol Use Disorders Identification Test- Consumption, *PHQ* Patient Health Questionnaire, *PSS* Perceived Stress Scale, *LOT-R* Life Orientation Test-Revised, *GASE* Generic Assessment of Side Effects. Hot flushes are assessed ambulatory via the hot flush diary. *At baseline, Health-related quality of life and expectations are assessed before and after the allocation, respectively. † The fourth study visit and the 8-week follow-up assessment takes place for the OLP 8wk and OLP 4wk groups only. ‡ For the 2nd allocation, the OLP group is further divided into the OLP 8wk and the OLP 4wk group. § The interview is conducted at week 8 or later.

Study sample

Eligible women are required to (1) experience at least 5 moderate or severe hot flushes per day, including night time, (2) with at least moderate ratings of bother (sum score ≥ 16 on the bother subscale of the Hot Flush Rating Scale (39)), (3) be fluent in German language, and (4) be in the menopausal transition (irregularities ≥ 60 days in the past year), or postmenopausal (cessation of menstruation ≥ 1 year) (40). Exclusion criteria are use of hormonal therapy, herbal remedies to treat hot flushes, or intake of selective serotonin reuptake inhibitor (SSRI)/ serotonin norepinephrine reuptake inhibitor (SNRI) within the last 6 weeks before enrolment, previous oophorectomy, severe physical or cognitive impairments which would constitute a barrier to give informed consent, severe depression or anxiety (≥ 9 sum score or ≥ 5 depression or anxiety subscore on the Patient Health Questionnaire (PHQ) -4) (41), and medical conditions which might cause hot flushes such as untreated hyperthyroidism, alcohol abuse (≥4 on AUDIT-C) (42) and cancer. After 8 weeks follow-up, four patients of each the OLP 4wk and the OLP 8wk group who indicated symptom improvement are invited to take part in the qualitative interview (N = 8).

Power analysis

The power calculation is based on our primary outcome hot flush score. The software G*Power was used to calculate the sample size a priori (43). Since no OLP study has been conducted in the field of hot flushes, we base our sample size calculation on two separate clusters of information. From double-blind hot flush trials, we can expect a moderate effect size of *Cohen's d* = 0.40 given a mean of 19, standard deviations of 10 (23), and a 4-point reduction in the hot flush score (frequency x severity) after 4 weeks of placebo (44). Based on previous OLP trials in patients with irritable bowel syndrome and chronic low back pain, a large effect size of d = 0.80 can be expected (28, 30). Combining these two clusters of information, a moderate to large effect size is expected for the difference between the OLP and the no-treatment group at post-treatment. For a two-tailed Student's t-test with an α-error rate of 0.05, a number of N = 90 participants would provide an 80% power to detect an effect of d = 0.60. We assumed an attrition rate of 10% and obtained our required sample size of N = 100 women.

Setting

This trial is conducted at the Psychosomatic Institute and Outpatients Clinic of the University Medical Center Hamburg-Eppendorf in Hamburg, Germany. Women are recruited through physician referrals, advertisements in newspapers and the internet, and flyers distributed in the greater Hamburg area. The treatment is described as "novel mind-body treatment". Interested volunteers can contact the study team for more information, and when positively screened on the phone, schedule the first study visit. Participants receive a reimbursement for their time to complete the hot flush diary and further questionnaires at the study visits.

Procedures

Prior to enrolment, informed consent is obtained by all participants. The informed consent and all study visits are conducted by clinicians (M.D. or M.Sc. psychologists). Self-report questionnaires are completed at each study visit, accompanied by a blinded assessor (study assistant). The assessor also gives instructions about how to complete the hot flush diary. Confidentiality is secured by replacing the participant's identifying information with a number. All identifying information is stored separately from the data. The file which links the number to the participant is stored locally; access is only granted to study clinicians and the principal investigator (PI). The file is deleted after the publication of results.

Clinicians are encouraged to use active listening during consultations, to avoid technical jargon and to devote equal attention, empathy, and interaction time to all participants, irrespective of allocation. A semi-scripting of the visits ensures both consistency and naturalness of patient-clinician interactions. We expect participants to vary in terms of their wish to talk about symptoms, skepticism towards the treatment, etc. Hence, we allow interaction times to differ up to 10 minutes for the first and up to 5 minutes for any other session. All information about the placebo treatment and the no-treatment group is provided prior to randomization and hence, equally to all participants irrespective of group allocation. This insures similar interactions with clinicians in both groups. Adverse events including worsening of symptoms are inquired at each study visit/call to facilitate clinician support and if necessary, initiate study discontinuation. All study visits (SV) and calls are outlined in the following.

SV 1 - Enrolment: Clinical interview and placebo briefing

After informed consent is signed, a clinical interview inquiring about hot flushes (duration, severity, bother) and related medical characteristics is conducted (see measures). Then, all patients are given a placebo briefing before randomization: (1) The placebo effect is powerful – placebos given under uncertain conditions, i.e., in clinical trials, have shown to produce significant alleviation of hot flushes. (2) Positive expectations might be helpful but are not necessary for the placebo to be effective. (3) The underlying mechanisms of OLP are unclear. Conditioning processes could constitute one of them. That is, the body might react to placebo pills in an automatic way since it has learned to associate the pill intake with symptom improvement. (4) Disbelief or doubts are fine but taking the pills faithfully is essential for the generation of a positive effect. Finally, (5) no OLP study has yet been conducted in the field of hot flushes. Hence, we encourage patients – if allocated to OLP in the following week - to "wait and see what will happen". Placebos are described as pills without pharmacologically active substances. To minimize stigmatizing associations of placebos (e.g. only gullible persons may benefit from placebos), we inform patients that placebos have been shown to produce measurable biochemical changes in the body. A short discussion about the importance of the no-treatment group follows. The session is concluded by informing the patient about further procedures. The first study visit takes about 30 to 40 minutes (time for questionnaire completion not included).

SV 2 - Baseline: Allocation

At the second study visit, patients are informed about their assignment by a clinician. Clinicians are not aware of the group assignment until the patient has opened the envelope and disclosed the allocation result. Clinicians instruct the OLP group to take 2 pills a day for 4 weeks, each morning and evening after the meal. A total number of 56 pills are handed out in a paper packaging which includes the pill bottle and the original medication leaflet of the producing company. The bottle shows the name of the pill ("placebo"), the number of pills and its equivalent in grams, a subtitle ("for menopausal hot flushes"), the contact data of the responsible party ("Department of Psychosomatic Medicine and Psychotherapy") and further instructions in view to the pills (e.g. "Store inaccessible to children"). Women are reminded about the importance of taking the pills faithfully and a 50%-chance of continuing the treatment for another 4 weeks.

Women assigned to the no-treatment group are reminded of the meaning of this study group. The duration of the second study visit is 5 to 10 minutes.

2-week call

The clinician asks about the occurrence of adverse events including aggravation of hot flushes. The OLP group is additionally inquired about treatment adherence and whether treatment has been interrupted.

SV 3 - Post-treatment: Second allocation (OLP group) and study conclusion (no-treatment group)

At the third study visit, all patients are inquired about their hot flushes and adverse events by the clinician. Patients in the OLP group are then assigned a second time; assignment results are disclosed by the clinician. Patients in both OLP 4wk and OLP 8wk groups are asked to complete the diary for another 4 weeks. The OLP 8wk group receives another bottle of placebos. For the no-treatment group, this constitutes the last study visit. Study diaries are collected, and women are thanked for their participation. The third study visit takes 10 to 15 minutes.

6-week call (OLP 4wk & OLP 8wk)

The same questions as in the 2-week call are asked in this 6-week call.

SV 4 - 8-week follow-up: Study conclusion (OLP 4wk & OLP 8wk)

Patients of the OLP 4wk and the OLP 8wk group are inquired about their hot flushes and adverse events by the clinician. Study diaries are collected, and women are thanked for their participation. The fourth study visit takes 10 to 15 minutes.

Qualitative interviews (subgroup of n = 8)

Shortly after the fourth study visit, a subgroup of participants is interviewed about their overall experiences with the treatment. The interview will be set up and analyzed with the method interpretative phenomenological analysis (IPA) which facilitates the understanding of subjective experiences and favors an in-depth exploration by using a loose agenda over a large number of participants (45). In alignment with the recommendations of the method, we aim to include a rather homogeneous sample. Thus, we will include only women who undergo the placebo treatment *and* indicate a perceived improvement, either at post-treatment or at 8-week follow-up. Of those patients, each one has a 50% chance to be invited to the interview, which is decided by drawing a card (yes/no). Informed consent is obtained separately from the quantitative part of the study, right before the scheduled interview. Ongoing enrolment is conducted until the full sample is reached. Themes include (1) how hot flushes affected everyday life before study participation, (2) prior experiences with hot flush treatments, (3) study motivation including expectations and hope regarding the treatment, (4) subjective explanation for improvement, and (5) symptom perception or change in symptom perception. The interview is recorded and transcribed verbatim.

Blinding

Due to the nature of the study, patients and clinicians are aware of group assignment. Assessors are blinded to group assignment of participants. To prevent the breaking of blinding, participants are requested to not communicate their group affiliation when they have questions about the assessments.

Randomization

Two randomizations are conducted. The first randomization takes place at baseline (second study visit) for all patients, and the second one takes place at post-treatment (third study visit) for the OLP group only. Both randomization sequences are generated prior to the first enrolment using an online program

(Sealed Envelope). A researcher who is otherwise not involved in the study notes the results of the allocation sequence and uses opaque, sealed, and sequentially numbered envelopes for its concealment. The sequences are then saved and locked away by the Principal Investigator (YN). Hence, it is not accessible to any person involved in the immediate study conduction. The allocation lists are stored in an office room of the department, inaccessible to blinded assessors throughout the study.

For the first randomization in which patients are 1:1 assigned to either OLP or no-treatment, we use permuted block randomization. The assignment is performed by the clinician at the beginning of the second study visit by opening the envelope.

For the second randomization, we use stratified permuted block randomization. Patients in the OLP group will be randomized 1:1 to OLP 4wk and OLP 8wk. The assignment is stratified for perceived improvement of hot flushes (yes/no). The clinician inquires the stratum and then performs the allocation.

Measures

Primary outcomes. The group difference between OLP and no-treatment in the hot flush composite score (frequency x severity), and bother by hot flushes at post-treatment are our primary outcomes. Women record number and severity of hot flushes in real-time, i.e., at occurrence, using a portable paper diary (25, 46). Night sweats are recorded on a subsequent morning. The diary is the gold standard for assessing hot flushes (47) and has shown high reliability and validity (25). In accordance with guideline recommendations, the severity of each hot flush is rated as mild, moderate, or severe (5). The severity categories are predefined which minimize divergence due to subjective evaluations. In the day, mild hot flushes are *not* accompanied by sweating. Moderate hot flushes are accompanied by sweating, whereas severe hot flushes lead to disruption of current activity. At night, mild hot flushes are spotted by damp sheets/clothing, whereas moderate, and severe hot flushes caused awakening. Severe hot flushes additionally necessitate actions like opening the window, removing sheets etc. The Hot Flush Rating Scale (39) assesses bother, i.e., to what extent hot flushes are regarded as problematic, distressing, and causing interference with daily life in the past week. Good reliability and validity are given for HFRS (39).

Secondary outcomes. These include the group difference between OLP and no-treatment in hot flush frequency, health-related quality of life, global improvement, and the number of responders (≥50% in hot flush frequency at 4 weeks from baseline) at post-treatment (48). Hot flush frequency is assessed as part of the diary. Health-related quality of life is measured with the Women’s Health Questionnaire (WHQ) (49), a questionnaire of proven reliability and validity (50) which covers eight domains including depressed mood, somatic symptoms, memory/concentration, vasomotor symptoms, anxiety, sexual life, sleep, and attractiveness. Although part of the questionnaire, the menstrual symptoms scale is excluded from analyses since many patients would be post-menopausal. Patients indicate their global improvement of hot flushes on a 7-point Likert scale (1 ‘very much worse’, 2 much worse’, 3 ‘minimally worse’, 4 ‘no change’, 5 ‘minimally improved’, 6 ‘much improved’, 7 ‘very much improved’) of the Patient Global Impression of Change scales (PGIC) (51). The PGIC is a validated and commonly used questionnaire in the field of pain and has been previously used in a sample with menopausal women (52).

Medical and sociodemographic characteristics, hot flush-related lifestyle and psychosocial variables. Medical characteristics are assessed via the clinical interview and include menopausal transition state, hysterectomy, years of hot flushes, previous intake of hot flush medication including experiences of benefits and adverse events, current body-mind interventions against hot flushes, and baseline symptoms. Usage of psychotherapy, body mass index and menopausal symptoms (Menopause Rating Scale-II) (53) are assessed via self-report questionnaires. Demographic variables include age, marital and occupational status, and educational level. Lifestyle variables include smoking, exercise (modified after the Menopause Health Questionnaire) (54) and alcohol consumption (AUDIT-C) (42). Perceived stress (PSS-10) (55, 56)

was shown to be associated with frequency, severity, and duration of hot flushes and might thus a potential cofounder of the treatment effect (2, 57, 58).

Expectations. Participants' (1) value expectations or hopes/wishes/desires about the treatment, (2) probability expectations, and (3) dispositional expectations, i.e., optimism, are assessed. Due to the lack of validated questionnaires in the field of expectations (59), we created two items (value expectations: "What change would you like to happen to your hot flushes over the next 4 weeks?"; probability expectations: "How do you expect your hot flushes to change over the next 6 weeks?") which are rated on a scale from 0 (*no change*) to 10 (*maximum improvement*). These items are based on a qualitative interview study with lower back pain patients (60). We assess optimism with the Life Orientation Test-Revised (LOT-R) (61). Optimism might constitute a predictor of placebo response, although evidence remains mixed (62, 63).

Adverse events. Adverse events are assessed openly and using a questionnaire. The list of symptoms in the self-report questionnaire includes loss of appetite, dry mouth, sleeping problems, nausea, dizziness, fatigue, constipation, nervousness, mood changes, and blank spaces for the specification of further symptoms. Severity (0 = not present, 1 = mild, 2 = moderate, 3 = severe) is rated for each symptom within the validated format of the GASE scale (64). To discern whether adverse events may be related to the treatment, the same list of symptoms is also inquired at baseline.

Adherence and treatment interruption. Intake-related information is assessed at week 2, 4, 6 and 8. Adherence is assessed via self-report with a single item ("How many placebo pills have you actually taken during the last week?"), which has been validated in a previous study with breast cancer patients (65). Treatment interruption is assessed through open-ended questions ("Did you interrupt the treatment? If yes, for how many days?").

Opinion about OLP usage. The question "Do you think OLP prescription in clinical practice is acceptable?" has been asked in a previous telephone survey study and is to be answered on a scale from 1 ('definitely yes') to 4 ('definitely not') (66).

Perceived improvement. The item "Have your hot flushes improved in the last 4 weeks?" (yes/no) is used as a stratum for the second randomization and as an indicator ("yes" as a requirement) for the qualitative interview.

Data management

All data collection procedures are semi-manualized. To reduce errors in data entry, every item answer is labeled with the original questionnaire score. After data entry, 10% of the data is controlled. If the number of mistakes exceeds 5% per case, another 10% of the data is controlled etc. Plausibility checks are conducted before the statistical analyses.

Statistical analyses

All hypotheses are tested two-sided with an α-level of 0.05.

Missing data. To analyze diary data, we aggregate daily data so that each data point comprises a weekly mean (Figure 2). Means are calculated if at least 3 days of the week are completed. Almost identical means of the hot flush diary were found for 3 and 7 days (67). Missing data points are not replaced since in linear mixed models, missing outcome data are handled using maximum likelihood estimation, assuming that data are missing at random conditional on information in the model. Single missing values in questionnaires are substituted by the mean of the remaining items, provided that 80% are completed (68).

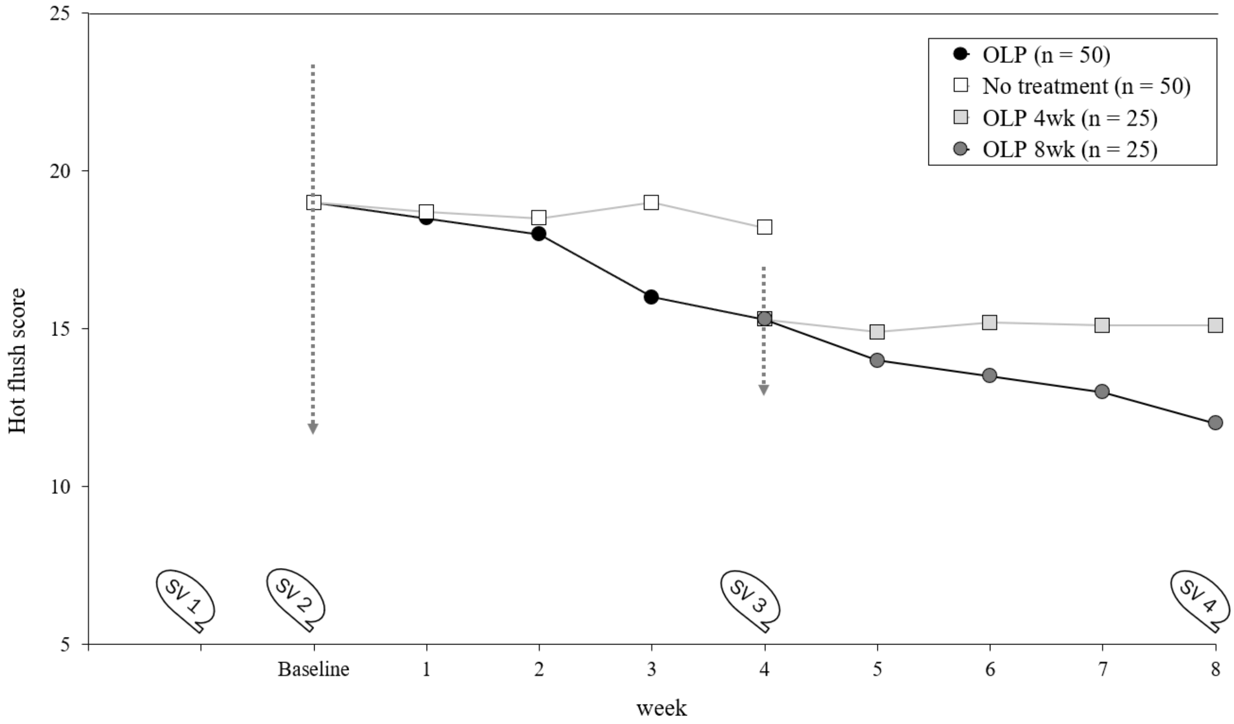

Figure 2. Study design with hypothesized trends of hot flush score. SV = study visit; OLP = open-label placebo; wk = week. The arrows represent the point in time of the first and second allocation. Circles represent placebo intake, squares represent no placebo intake. OLP 4wk and OLP 8wk are subgroups of the OLP group.

Efficacy. For our primary outcome hot flush composite score, we conduct a linear mixed model for repeated measures using restricted maximum likelihood estimations with the intent-to-treat sample. We define group membership (OLP / no treatment), measurement point, and their interaction term as fixed effects. The baseline score is additionally included as a covariate. Measurement points are treated as repeated measures with an autoregressive residual covariance structure. Moreover, the model includes a random intercept to model between-subject differences. A normal distribution with an identity link is used. Since each data point reflects a weekly mean, up to 5 weekly means are included for each participant. Effect sizes are obtained by dividing the estimated group difference by the product of standard error by square rooted number of participants in the no-treatment group (69). For our primary outcome bother of hot flushes, we will perform a linear mixed model with two data points at baseline and post-treatment. A post-hoc test comparing OLP with no-treatment will be conducted to test the group difference at post-treatment.

By fitting (generalized) linear mixed models, the analytical strategy for all secondary outcomes corresponds with the one described for the primary outcome. For metric outcomes (health-related quality of life and global improvement) we use a normal distribution with an identity link. For dichotomous outcomes (response), we use a binomial distribution with a logit link, and for count outcomes (hot flush frequency), we use a negative binomial distribution with a log-link.

Sensitivity analyses. Sensitivity analyses include rerun of outcome analyses with missing values substituted using the last-observation-carried-forward method, per-protocol analyses (exclusion of patients who have discontinued treatment or reported adherence rates <90%), analyses with adjustment for the variables ‘years of hot flushes’, and stress, and after exclusion of patients who have conducted lifestyle changes in the course of the study.

Further research questions

Duration. To investigate whether a prolonged intake facilitates increased symptom improvements, linear mixed models which align with the ones of the efficacy question are performed to examine differences between the groups OLP 4wk and OLP 8wk. With *n* = 25 participants per group, significant effects would be present if the effect size was *Cohen's d* = 0.8. Since no study has investigated the duration of OLP in a randomized-controlled design, we are unaware whether an effect exists at all. Hence, the comparison between the two OLP groups is exploratory and the study not deliberately powered for this sub-analysis.

Maintenance. To test whether beneficial effects can be sustained after the intake is stopped, we perform contrast analyses within the OLP 4wk group with the pre-defined contrasts (A) 'baseline' vs. '4 wk', (B) 'baseline vs. '8 wk' and (C) '4 wk' vs. '8 wk'. If sustained efficacy is given, contrasts (A) and (B) would be statistically significant, whereas contrast (C) would be non-significant. Aligned with the "duration" question, this question is also an exploratory one.

Adverse events. To investigate treatment safety, we will perform an exploratory comparison of rates of adverse events between the OLP group and the no-treatment group. Individual baseline symptoms will be considered when interpreting the results.

Role of expectations. To investigate whether positive expectations amplify beneficial effects of the OLP treatment, value expectations, probability expectations, and optimism are separately included in the primary outcome models as candidate moderators. The interaction effect time x group x expectation variable will be tested with significant effects indicating that expectation moderates group effects. The trajectory of value and probability expectations over time will also be subject to exploratory analysis.

Qualitative analysis. According to IPA procedures, transcripts are analyzed line-by-line to find emerging themes of each participant. Themes are then clustered within patients and compared between patients. The first two interviews will be transcribed and analyzed recently after execution by two researchers. Notes and themes are compared; discrepancies are discussed. Further interviews are analyzed by the researchers independently.

List of abbreviations

ADHD: Attention deficit hyperactivity disorder; AUDIT-C: Alcohol Use Disorders Identification Test-Consumption; BMI: Body Mass Index; CAM: Complementary and alternative medicine; GASE: Generic Assessment of Side Effects; HFRS: Hot Flush Rating Scale; IPA: Interpretative phenomenological analysis; LOT-R: Life Orientation Test-Revised; MHQ: Menopause Health Questionnaire; MRS: Menopause Rating Scale; OLP: Open-label placebo; PGIC: Patient Global Impression of Change; PHQ: Patient Health Questionnaire; PI: Principal investigator; PSS: Perceived Stress Scale; SSRI: Selective serotonin reuptake inhibitor; SNRI: Serotonin norepinephrine reuptake inhibitor; SV: study visit; WHQ: Women's Health Questionnaire

Access to data. The PI (YN) and her research team has full access to data.

Dissemination policy. The results of the trial will be published. No restrictions apply.

REFERENCES

1. Freeman EW, Sammel MD, Sanders RJ. Risk of long-term hot flashes after natural menopause: evidence from the Penn Ovarian Aging Study cohort. Menopause. 2014;21(9):924-32.

2. Avis NE, Crawford SL, Greendale G, Bromberger JT, Everson-Rose SA, Gold EB, et al. Duration of menopausal vasomotor symptoms over the menopause transition. JAMA Intern Med. 2015;175(4):531-9.
3. Williams RE, Levine KB, Kalilani L, Lewis J, Clark RV. Menopause-specific questionnaire assessment in US population-based study shows negative impact on health-related quality of life. Maturitas. 2009;62(2):153-9.
4. Dibonaventura MD, Chandran A, Hsu MA, Bushmakin A. Burden of vasomotor symptoms in France, Germany, Italy, Spain, and the United Kingdom. Int J Womens Health. 2013;5(5):261-9.
5. European Medicines Agency. Guideline on clinical investigation of medicinal products for hormone replacement therapy of oestrogen deficiency symptoms in postmenopausal women. 2005. http://www.ema.europa.eu/docs/en_GB/document_library/scientific_guideline/2009/09/WC500003348.pdf. Accessed 22 Apr 2019.
6. Maclennan AH, Broadbent JL, Lester S, Moore V. Oral oestrogen and combined oestrogen/progestogen therapy versus placebo for hot flushes. The Cochrane Database of Systematic Reviews. 2004(4):CD002978-CD.
7. Grant M, Marbella A, Wang A, Pines E, Hoag J, Bonnell C, et al. Menopausal Symptoms: Comparative Effectiveness of Therapies. Rockville: Agency for Healthcare Research and Quality (US); 2015. Report No.: 15-EHC005-EF.
8. Heiss G, Wallace R, Anderson GL, Aragaki A, Beresford SA, Brzyski R, et al. Health risks and benefits 3 years after stopping randomized treatment with estrogen and progestin. JAMA. 2008;299(9):1036-45.
9. Hulley S, Grady D, Bush T, Furberg C, Herrington D, Riggs B, et al. Randomized trial of estrogen plus progestin for secondary prevention of coronary heart disease in postmenopausal women. Heart and Estrogen/progestin Replacement Study (HERS) Research Group. JAMA. 1998;280(7):605-13.
10. Sprague BL, Trentham-Dietz A, Cronin KA. A sustained decline in postmenopausal hormone use: results from the National Health and Nutrition Examination Survey, 1999-2010. Obstet Gynecol. 2012;120(3):595-603.
11. The North American Menopause Society. Nonhormonal management of menopause-associated vasomotor symptoms: 2015 position statement of The North American Menopause Society. Menopause. 2015;22(11):1155-72.
12. Nelson HD, Vesco KK, Haney E, Fu R, Nedrow A, Miller J, et al. Nonhormonal therapies for menopausal hot flashes: systematic review and meta-analysis. JAMA. 2006;295(17):2057-71.
13. Brett KM, Keenan NL. Complementary and alternative medicine use among midlife women for reasons including menopause in the United States: 2002. Menopause. 2007;14(2):300-7.
14. van der Sluijs CP, Bensoussan A, Liyanage L, Shah S. Women's health during mid-life survey: the use of complementary and alternative medicine by symptomatic women transitioning through menopause in Sydney. Menopause. 2007;14(3):397-403.
15. Buhling KJ, Daniels BV, Studnitz FS, Eulenburg C, Mueck AO. The use of complementary and alternative medicine by women transitioning through menopause in Germany: results of a survey of women aged 45-60 years. Complement Ther Med. 2014;22(1):94-8.
16. Franco OH, Chowdhury R, Troup J, Voortman T, Kunutsor S, Kavousi M, et al. Use of Plant-Based Therapies and Menopausal Symptoms: A Systematic Review and Meta-analysis. JAMA. 2016;315(23):2554-63.

17. Drewe J, Bucher KA, Zahner C. A systematic review of non-hormonal treatments of vasomotor symptoms in climacteric and cancer patients. Springerplus. 2015;4(65):65.

18. Borrelli F, Ernst E. Alternative and complementary therapies for the menopause. Maturitas. 2010;66(4):333-43.

19. Zhu X, Liew Y, Liu ZL. Chinese herbal medicine for menopausal symptoms. Cochrane Database Syst Rev. 2016;3:CD009023.

20. Dodin S, Blanchet C, Marc I, Ernst E, Wu T, Vaillancourt C, et al. Acupuncture for menopausal hot flushes. Cochrane Database Syst Rev. 2013(7):CD007410.

21. Saensak S, Vutyavanich T, Somboonporn W, Srisurapanont M. Relaxation for perimenopausal and postmenopausal symptoms. Cochrane Database Syst Rev. 2014(7):CD008582.

22. Elkins GR, Fisher WI, Johnson AK, Carpenter JS, Keith TZ. Clinical hypnosis in the treatment of postmenopausal hot flashes: a randomized controlled trial. Menopause. 2013;20(3):291-8.

23. Carmody JF, Crawford S, Salmoirago-Blotcher E, Leung K, Churchill L, Olendzki N. Mindfulness training for coping with hot flashes: results of a randomized trial. Menopause. 2011;18(6):611-20.

24. Ayers B, Smith M, Hellier J, Mann E, Hunter MS. Effectiveness of group and self-help cognitive behavior therapy in reducing problematic menopausal hot flushes and night sweats (MENOS 2): a randomized controlled trial. Menopause. 2012;19(7):749-59.

25. Sloan JA, Loprinzi CL, Novotny PJ, Barton DL, Lavasseur BI, Windschitl H. Methodologic lessons learned from hot flash studies. J Clin Oncol. 2001;19(23):4280-90.

26. Freeman EW, Ensrud KE, Larson JC, Guthrie KA, Carpenter JS, Joffe H, et al. Placebo improvement in pharmacologic treatment of menopausal hot flashes: time course, duration, and predictors. Psychosom Med. 2015;77(2):167-75.

27. Loprinzi CL, Sloan J, Stearns V, Slack R, Iyengar M, Diekmann B, et al. Newer antidepressants and gabapentin for hot flashes: an individual patient pooled analysis. J Clin Oncol. 2009;27(17):2831-7.

28. Carvalho C, Caetano JM, Cunha L, Rebouta P, Kaptchuk TJ, Kirsch I. Open-label placebo treatment in chronic low back pain: a randomized controlled trial. Pain. 2016;157(12):2766-72.

29. Kam-Hansen S, Jakubowski M, Kelley JM, Kirsch I, Hoaglin DC, Kaptchuk TJ, et al. Altered placebo and drug labeling changes the outcome of episodic migraine attacks. Sci Transl Med. 2014;6(218):218ra5.

30. Kaptchuk TJ, Friedlander E, Kelley JM, Sanchez MN, Kokkotou E, Singer JP, et al. Placebos without deception: A randomized controlled trial in irritable bowel syndrome. PloS one. 2010;5(12):e15591-e.

31. Kelley JM, Kaptchuk TJ, Cusin C, Lipkin S, Fava M. Open-label placebo for major depressive disorder: A pilot randomized controlled trial. Psychotherapy and Psychosomatics. 2012;81(5):312-4.

32. Schaefer M, Harke R, Denke C. Open-Label Placebos Improve Symptoms in Allergic Rhinitis: A Randomized Controlled Trial. Psychother Psychosom. 2016;85(6):373-4.

33. Schaefer M, Sahin T, Berstecher B. Why do open-label placebos work? A randomized controlled trial of an open-label placebo induction with and without extended information about the placebo effect in allergic rhinitis. PLoS One. 2018;13(3):e0192758.

34. Locher C, Frey Nascimento A, Kirsch I, Kossowsky J, Meyer A, Gaab J. Is the rationale more important than deception? A randomized controlled trial of open-label placebo analgesia. Pain. 2017;158(12):2320-8.

35. Mathur A, Jarrett P, Broadbent E, Petrie KJ. Open-label Placebos for Wound Healing: A Randomized Controlled Trial. Annals of Behavioral Medicine. 2018.
36. Hoenemeyer TW, Kaptchuk TJ, Mehta TS, Fontaine KR. Open-Label Placebo Treatment for Cancer-Related Fatigue: A Randomized-Controlled Clinical Trial. Sci Rep. 2018;8(1):2784.
37. Locher C, Frey Nascimento A, Kirsch I, Kossowsky J, Meyer A, Gaab J. Is the rationale more important than deception? A randomized controlled trial of open-label placebo analgesia. Pain. 2017.
38. Chan AW, Tetzlaff JM, Gotzsche PC, Altman DG, Mann H, Berlin JA, et al. SPIRIT 2013 explanation and elaboration: guidance for protocols of clinical trials. BMJ. 2013;346:e7586.
39. Hunter MS, Liao KL. A psychological analysis of menopausal hot flushes. Br J Clin Psychol. 1995;34(4):589-99.
40. Harlow SD, Gass M, Hall JE, Lobo R, Maki P, Rebar RW, et al. Executive summary of the Stages of Reproductive Aging Workshop + 10: addressing the unfinished agenda of staging reproductive aging. J Clin Endocrinol Metab. 2012;97(4):1159-68.
41. Kroenke K, Spitzer RL, Williams JB, Lowe B. An ultra-brief screening scale for anxiety and depression: the PHQ-4. Psychosomatics. 2009;50(6):613-21.
42. Bush K, Kivlahan DR, McDonell MB, Fihn SD, Bradley KA. The AUDIT Alcohol Consumption Questions (AUDIT-C). An Effective Brief Screening Test for Problem Drinking. Archives of Internal Medicine. 1998;158(16):1789.
43. Faul F, Erdfelder E, Lang AG, Buchner A. G*Power 3: a flexible statistical power analysis program for the social, behavioral, and biomedical sciences. Behav Res Methods. 2007;39(2):175-91.
44. Stearns V, Beebe KL, Iyengar M, Dube E. Paroxetine controlled release in the treatment of menopausal hot flashes: a randomized controlled trial. JAMA. 2003;289(21):2827-34.
45. Smith JA, Flowers P, Larkin M. Interpretative Phenomenological Analysis: Theory, Method and Research. London: SAGE Publications; 2009.
46. Guttuso T, Jr., DiGrazio WJ, Reddy SY. Review of hot flash diaries. Maturitas. 2012;71(3):213-6.
47. Miller HG, Li RM. Measuring hot flashes: summary of a National Institutes of Health workshop. Mayo Clin Proc. 2004;79(6):777-81.
48. Butt DA, Deng LY, Lewis JE, Lock M. Minimal decrease in hot flashes desired by postmenopausal women in family practice. Menopause. 2007;14(2):203-7.
49. Hunter MS. The women's health questionnaire: A measure of mid-aged women's perceptions of their emotional and physical health. Psychology & Health. 1992;7(1):45-54.
50. Shin H, Shin HS. Measurement of quality of life in menopausal women: a systematic review. West J Nurs Res. 2012;34(4):475-503.
51. Geisser ME, Clauw DJ, Strand V, Gendreau RM, Palmer R, Williams DA. Contributions of change in clinical status parameters to Patient Global Impression of Change (PGIC) scores among persons with fibromyalgia treated with milnacipran. Pain. 2010;149(2):373-8.
52. Guttuso T, Jr., Kurlan R, McDermott MP, Kieburtz K. Gabapentin's effects on hot flashes in postmenopausal women: a randomized controlled trial. Obstet Gynecol. 2003;101(2):337-45.
53. Hauser GA, Potthoff P, Rosemeier PJ, Schneider HPG. Die Selbstbeurteilungs-Skala für klimakterische Beschwerden (Menopause Rating Scale II). Journal für Menopause. 1999;4.
54. The North American Menopause Society. Menopause Health Questionnaire. Pepper Pike, OH, USA; 2017.

55. Cohen S, Kamarck T, Mermelstein R. A global measure of perceived stress. J Health Soc Behav. 1983;24(4):385-96.
56. Klein EM, Brahler E, Dreier M, Reinecke L, Muller KW, Schmutzer G, et al. The German version of the Perceived Stress Scale - psychometric characteristics in a representative German community sample. BMC Psychiatry. 2016;16:159.
57. Hunter MS, Chilcot J. Testing a cognitive model of menopausal hot flushes and night sweats. J Psychosom Res. 2013;74(4):307-12.
58. Worsley R, Bell R, Kulkarni J, Davis SR. The association between vasomotor symptoms and depression during perimenopause: a systematic review. Maturitas. 2014;77(2):111-7.
59. Laferton JA, Kube T, Salzmann S, Auer CJ, Shedden-Mora MC. Patients' Expectations Regarding Medical Treatment: A Critical Review of Concepts and Their Assessment. Front Psychol. 2017;8:233.
60. Haanstra TM, Hanson L, Evans R, van Nes FA, De Vet HC, Cuijpers P, et al. How do low back pain patients conceptualize their expectations regarding treatment? Content analysis of interviews. Eur Spine J. 2013;22(9):1986-95.
61. Hinz A, Sander C, Glaesmer H, Brahler E, Zenger M, Hilbert A, et al. Optimism and pessimism in the general population: Psychometric properties of the Life Orientation Test (LOT-R). International Journal of Clinical and Health Psychology. 2017;17(2):161-70.
62. Geers AL, Wellman JA, Fowler SL, Helfer SG, France CR. Dispositional optimism predicts placebo analgesia. J Pain. 2010;11(11):1165-71.
63. Pecina M, Azhar H, Love TM, Lu T, Fredrickson BL, Stohler CS, et al. Personality trait predictors of placebo analgesia and neurobiological correlates. Neuropsychopharmacology. 2013;38(4):639-46.
64. Rief W, Glombiewski JA, Barsky AJ. Generic Assessment of Side Effects: GASE Bern: Verlag Hans Huber; 2009 [cited 2019 April 22]. Available from: http://www.gase-scale.com/.
65. Ziller V, Kalder M, Albert US, Holzhauer W, Ziller M, Wagner U, et al. Adherence to adjuvant endocrine therapy in postmenopausal women with breast cancer. Ann Oncol. 2009;20(3):431-6.
66. Hull SC, Colloca L, Avins A, Gordon NP, Somkin CP, Kaptchuk TJ, et al. Patients' attitudes about the use of placebo treatments: telephone survey. BMJ. 2013;347:f3757.
67. Grady D, Macer J, Kristof M, Shen H, Tagliaferri M, Creasman J. Is a shorter hot flash diary just as good as a 7-day diary? Menopause. 2009;16(5):932-6.
68. Bell ML, Fairclough DL, Fiero MH, Butow PN. Handling missing items in the Hospital Anxiety and Depression Scale (HADS): a simulation study. BMC Res Notes. 2016;9(1):479.
69. Feingold A. Effect sizes for growth-modeling analysis for controlled clinical trials in the same metric as for classical analysis. Psychol Methods. 2009;14(1):43-53.
